# Supplementary material for: AP-1 mediates cellular adaptation and memory formation
Source: Nat Commun. 2026 Mar 20;17:4265. doi: 10.1038/s41467-026-70862-w (PMC13168276; doi:10.1038/s41467-026-70862-w)
Supplement: Supplementary file 1 — Supplemental information [file 41467_2026_70862_MOESM1_ESM.pdf]

Supplemental Information

Supplemental Figures and Figure Legends

Supplemental Figure 1. Accessibility changes in peaks as cells transition from primed to adapted to resistant.

Supplemental Figure 2. Additional evidence for cellular adaptation during the acquisition of therapy resistance

Supplemental Figure 3: Extended evidence shows cellular adaptation in therapy resistance is dependent on AP-1

Supplemental Figure 4. Adaptation and memory also observed in H358 cell line of non-small cell lung cancer

Supplemental Figure 5. Validation of dexamethasone-induction and reversion of gene expression in naive and resistant cells

Supplemental Figure 6. Validation of AP-1 inhibition by JNK inhibition and comprehensive chromatin accessibility analysis supports an AP-1-dependent mechanism of memory formation.

Supplemental Figure 7. Validation of the dynamics, stability, and specificity of AP-1 reporter cells

Supplemental Figure 8. Validation of the dynamics of a second clone of AP-1 reporter cells - WM989-A6-G3-CC10

Supplemental Figure 9. Gating strategy for barcoded (GFP+) cell lines used in FateMap experiments

Tables

Supplementary Table 1: Cloning primers for generation of AP-1 responsive fluorescent reporters

Supplementary Table 2: Primers used to generate gDNA barcode sequencing libraries

Supplementary Table 3: Sequences of the smFISH probes used in this study

Supplementary Table 4: Primers used to index ATAC sequencing libraries

Extended Statistical Information

Supplemental Figures and Figure Legends

Supplemental Figure 1

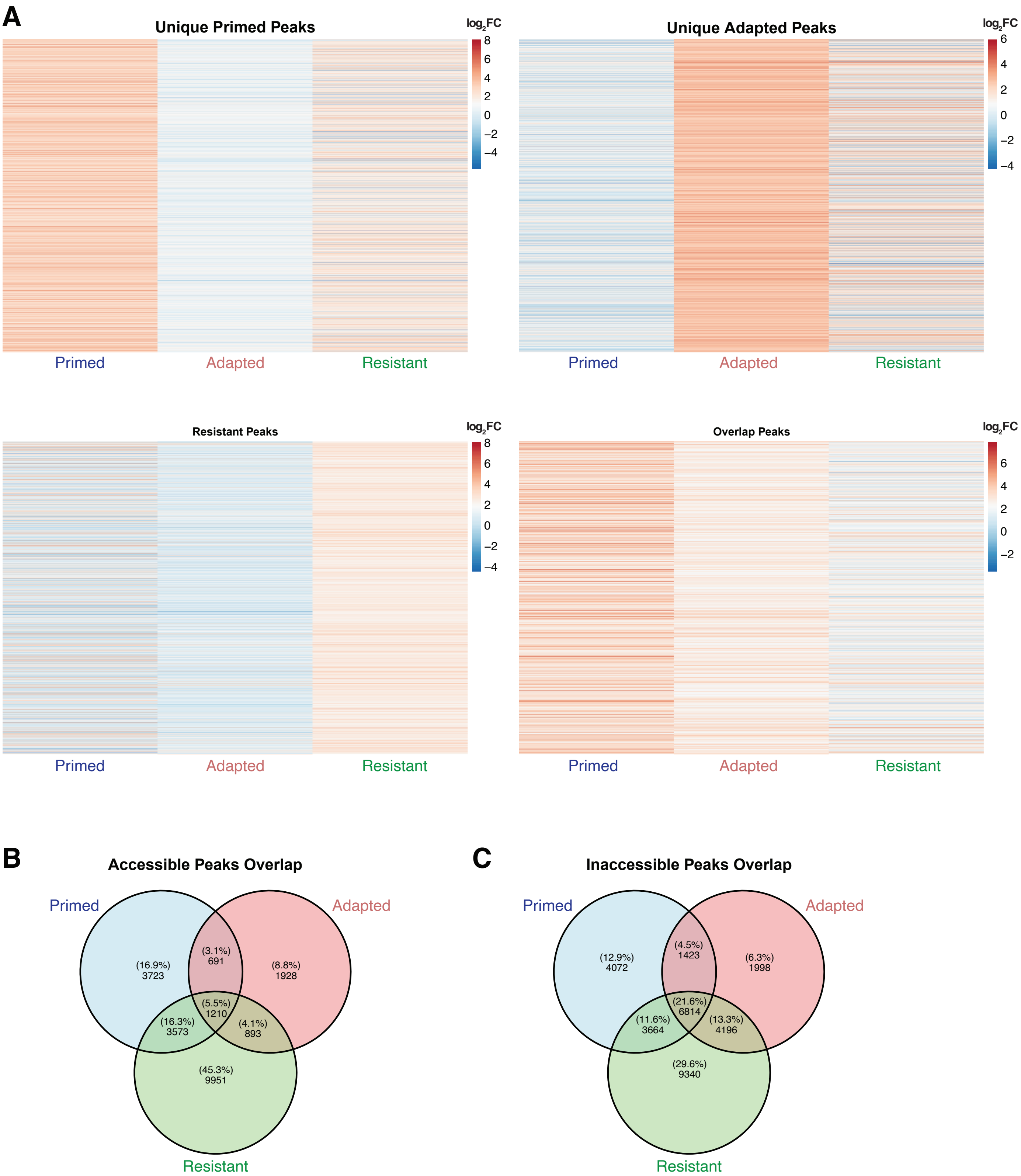

Supplemental Figure 1. Accessibility changes in peaks as cells transition from primed to adapted to resistant.

(A) Heatmaps showing log2 fold change patterns across primed, adapted and resistant conditions for four distinct peak sets: unique primed peaks (peaks with increased accessibility only in primed cells compared to adapted), unique adapted peaks (peaks with increased accessibility only in adapted cells compared to primed cells), resistant peaks (peaks with increased accessibility in resistant cells), and overlap peaks (peaks with increased accessibility shared between primed and adapted cells.

(B) Venn diagram of peak overlap between peaks that have gained accessibility in EGFR+ WM989 A6-G3 cells before drug addition (primed cells), 1 week after drug (adapted cells), and 4 weeks after drug addition (resistant cells. Peaks were defined as increased accessibility if they had a log2(fold change) greater than 1 and adjusted p-value less than 0.05 compared to EGFR-negative cells without drug.

(C) Venn diagram of peak overlap between peaks that have lost accessibility in EGFR+ WM989 A6-G3 cells before drug addition (primed cells), 1 week after drug (adapted cells), and 4 weeks after drug addition (resistant cells. Peaks were defined as decreased accessibility if they had a log2 (fold change) less than -1 and adjusted p-value less than 0.05 compared to EGFR-negative cells without drug.

To answer the question as to whether there are specific clusters of loci becoming accessible only following adaptation versus clusters that become accessible already in the primed state, we performed a peak-centric analysis on the ATAC dataset from Figure 1. We classified the conditions as EGFR negative (not primed), EGFR positive no drug (primed), EGFR positive 1 week of drug (adapted), and EGFR positive 4 weeks of drug (resistant). We then compared the peaks that have increased accessibility compared to not primed and checked which ones were consistent across conditions and which were unique to particular ones **(Supplemental Figure 1A)**. We found that 5.5% of the peaks were already more accessible in the primed condition and stayed open all the way through resistance, while many other peaks were unique to particular conditions. Interestingly, there was a higher degree of overlap between primed and resistant (16.3%) as opposed to primed and adapted (3.1%) or resistant and adapted (4.1%) **(Supplemental Figure 1B)**. We also identified peaks from primed, adapted, and resistant conditions and found ones that lose accessibility compared to not primed cells **(Supplemental Figure 1C)**. We found that of these peaks, 21.6% were already lost in primed and remained inaccessible throughout resistance. Again, we found many peaks that were unique to each of the conditions, with 29.6% of these lost peaks unique to the resistant cells.

Supplemental Figure 2

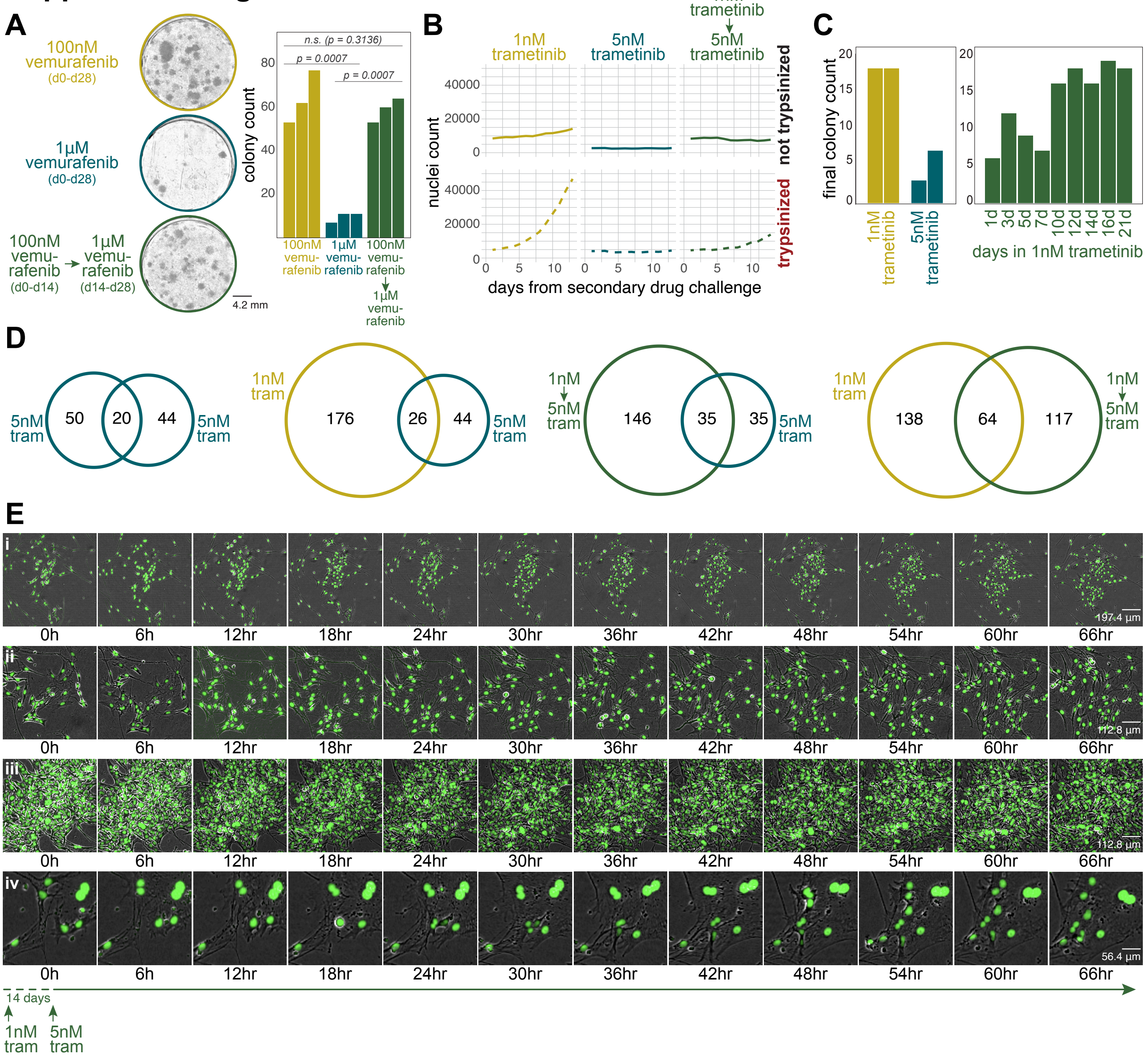

Supplemental Figure 2. Additional evidence for cellular adaptation during the acquisition of therapy resistance
(A) Vemurafenib dose-escalation experiment: Representative images of WM989 A6-G3 colonies at day 28 for low dose alone, high dose alone, and low-to-high-dose switch conditions. Bar plots show average resistant colony counts from 3 biological replicates at the experiment end point. Statistical analysis: Shapiro-Wilk test for normality of residuals, Levene's test for variance homogeneity, and post-hoc pairwise comparisons using estimated marginal means with Holm correction for multiple comparisons
(B) Test of resistant colony density on trametinib dose-escalation response. Graph shows nuclei count over time, with day 0 representing the dose switch/re-plating day. Data was collected from the same sample measured repeatedly throughout the time course of the experiment (n=1).
(C) Additional time course of low-to-high-dose switch (24-well format) (n=1): Left: Bar plots comparing resistant colony numbers under continuous low versus high dose conditions (two replicates for each condition). Right: Bar plots showing resistant colony numbers when switching to high dose after varying low dose exposure durations. Measurements were taken from distinct samples.
(D) Venn diagrams of barcode overlap in DNA barcode lineage tracing experiments for low-dose, high-dose alone, and low-to-high-dose switch conditions. (left to right) high dose condition compared with a technical replica of high dose condition from the same experiment, low dose condition compared with high dose condition, low-to-high-dose switch condition compared with high dose condition, low dose condition compared with low-to-high-dose switch condition (n=1).
(E) High-resolution time-lapse imaging of colonies immediately following the switch from low to high dose. The top row shows images of the full colony from Figure 2F, while the lower panels show examples of three additional colonies. All colonies shown here developed in the presence of 1nM trametinib, with day 0 marking the start of the switch into high dose. Each row shows a single colony imaged repeatedly every 6 hours.

Since much of the initial characterization of this system[16] was done with vemurafenib, we first confirmed that the phenomenon of adaptation seen with trametinib is also replicated with BRAF^{V600E} inhibitor vemurafenib. As in the trametinib data, treatment with low-dose vemurafenib (100 nM) for 14 days prior to challenge with high-dose vemurafenib (1 μM) allowed for the survival of colonies that otherwise would not have survived in high-dose vemurafenib (1 μM). These data demonstrated that the phenomenon of adaptation also occurs with vemurafenib treatment **(Supplemental Figure 2A).** Next, to rule out the possibility that the increased ability of cells to survive high-dose trametinib (5 nM) is due to the increased cell density within the small colonies that formed by the time of the switch to high-dose trametinib, we compared the usual protocol of growing the cells in one well for the duration of the experiment to a protocol in which we trypsinized cells on the day of dose-switch and re-plated the same number of cells as were plated at the start of the experiment. As expected, we found that in splitting and re-plating cells, there was more notable growth by cell count as compared to growing the cells in one well throughout, presumably due to decreased contact inhibition. Additionally, amongst the trypsinized conditions, cells grown in low-dose for the first 14 days grew at a higher rate when replated and exposed to high-dose trametinib as compared to cells that have survived high-dose and then replated and exposed to the same high-dose trametinib. These data suggest that cell density does not protect cells during the low-to-high-dose switch **(Supplemental Figure 2B)**. As an additional control, we repeated the time course from Figure 2C in a 24-well plate format, and we still saw that it took about 12-16 days of low-dose treatment for the number of surviving colonies to plateau. These data provide further support that adaptation is time-dependent **(Supplemental Figure 2C)**.

In addition to quantifying the number of DNA lineage barcodes that survived across the various conditions from Fig. 2D. We also analyzed the overlap of specific barcodes across conditions; our analysis revealed barcodes that survived specifically in the low-to-high-dose switch condition but not in the high dose alone, suggesting that these cells adapted to the therapy during low dose exposure. We also observed that twins of cells surviving the low-to-high-dose switch condition were also likely to survive in the low-dose alone condition

(Supplemental Figure 2D). This finding supports the conclusion that the additional cells surviving at low dose are the ones that persist through the low-to-high-dose switch, rather than a subset of cells inherently resistant to high dose treatment.

As a complement to the time-lapse images in Figure 2F, high-resolution time-lapse imaging in **Supplemental Figure 2E** shows colonies during the switch from low to high dose (upper panels: full colony from Figure 2F, lower panel: another example colony). These time-lapse images of two representative colonies demonstrated several key points: 1. No notable cell death was occurring (which would have appeared as holes in the colony that might have been filled over days of additional cell division), which we would have expected to see if progressive selection was occurring within these colonies. 2. The images showed clear cell proliferation during the high-dose challenge. These observations rule out the possibility of further progressive selection within low-dose surviving colonies when subsequently challenged with high-dose trametinib.

Supplemental Figure 3

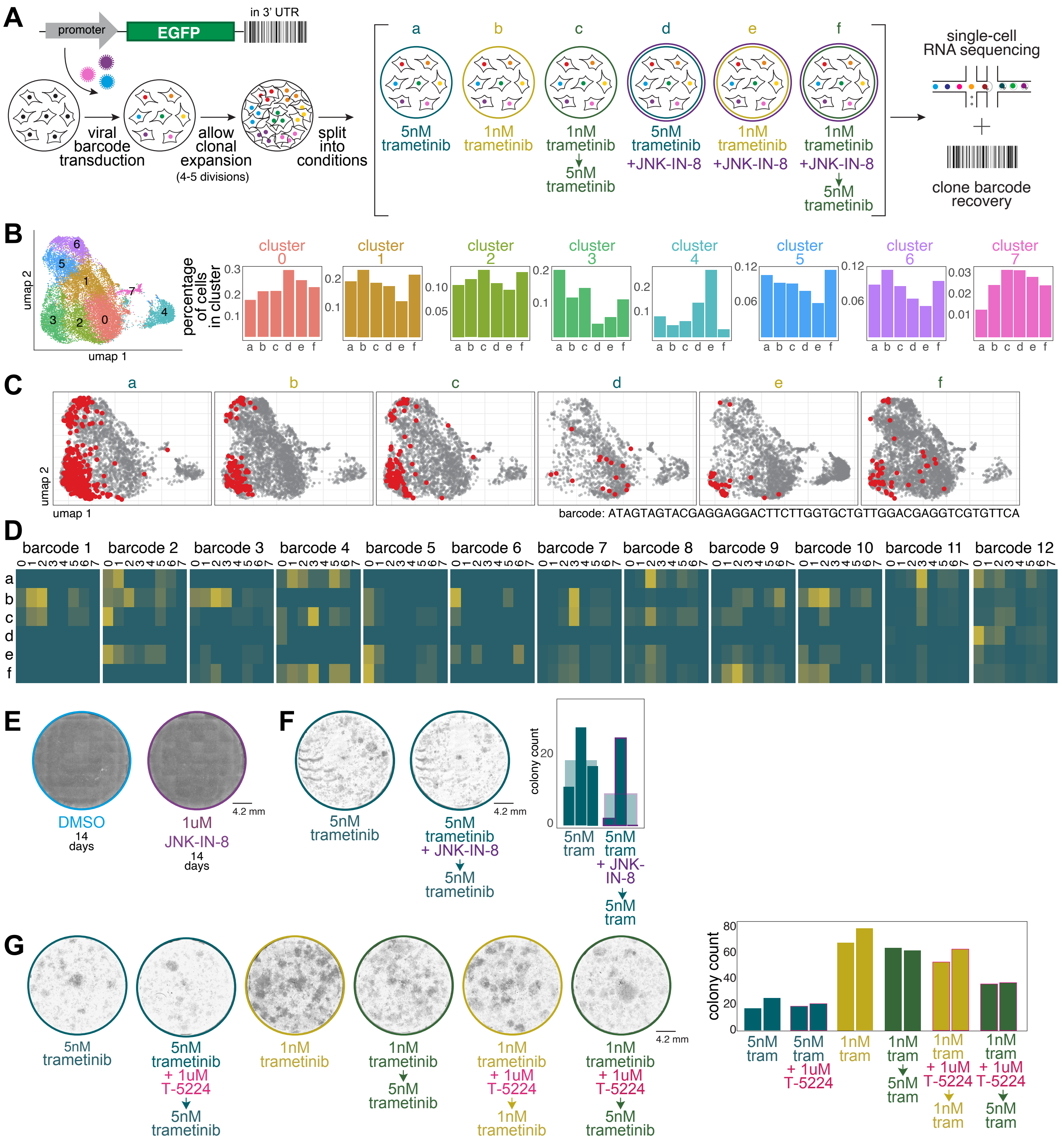

Supplemental Figure 3: Extended evidence shows cellular adaptation in therapy resistance is dependent on AP-1

(A) Schematic illustrating FateMap experiment assessing transcriptional heterogeneity across resistant lineages under different treatment conditions: WM989 A6-G3 cells were transduced with the FateMap barcode library (MOI ≈ 0.2). After 4–5 cell divisions, the barcoded population was sorted and split into illustrated conditions. Treated cells underwent single-cell RNA-seq and barcode sequencing (Table 4.3). Samples are designated as conditions a-f in subsequent panels.

(B) Left: UMAP showing cluster identities from all samples from the same scRNA sequencing experiment integrated using Harmony and clustered with Seurat. Right: Cluster ratio analysis depicting the percentage of cells from each condition in each cluster.

(C) UMAPs displaying the transcriptional states of representative twin clones (cells sharing the same barcode) across the six experimental conditions. The unique barcode sequence for this example is shown in the bottom right corner of each set of UMAPs.

(D) Heatmap of 12 example barcodes (lineages) in 12 separate blocks. Rows represent experimental conditions; columns represent clusters from panel G.

(E) Effect of JNK-IN-8 alone on the growth of therapy-naive cells after 14 days of treatment with either DMSO or 1 μM JNK-IN-8 (n=1).

(F) Effect of the 5 nM trametinib treatment alone compared to the effect of co-treatment of 5 nM trametinib with 1 μM JNK-IN-8. Left: Representative whole-well images from one of the three biological replicates. Right: Bar plot showing colony counts for 3 biological replicates.

(G) Effect of AP-1 inhibition using T-5224. Left: Representative whole-well images at day 14 (midpoint and time of dose-switch) and at day 28 (endpoint) showing colony formation under different treatment conditions from one of the two biological replicates. Right: Bar plot quantifying final colony counts for each condition. Each bar represents a biological replicate (n=2).

To complement the DNA lineage barcoding shown in Figure 2D, we combined lineage barcoding with single-cell RNA sequencing. This approach allowed us to compare transcriptional state changes in resistant colonies that emerged under the various treatment conditions described **(Supplemental Figure 3A)**. We integrated the reads from scRNAseq with Harmony. We visualized those clusters on a UMAP to ensure that colonies that survived in the low-to-high-dose switch condition were not in a completely new cell state that was not present in the low-dose or high-dose alone conditions. When we separated out each cluster identified via Seurat and compared, for each experimental condition, the percentage of the cells recovered in that condition fell into that cluster **(Supplemental Figure 3B).** Our analysis revealed no significant changes in cluster composition across conditions and no condition-specific clusters, suggesting that the transition from low to high-dose treatment does not induce entirely new cellular fates beyond those observed in either low or high-dose conditions alone. To examine the fate of twin clones (cells sharing the same barcode) across different conditions, we traced the locations of cells with a specific barcode on the UMAP for each condition. In this example, we observed that cells with the selected barcode showed similar cluster distributions in the low-dose, low-to-high-dose switch, and high-dose conditions. Notably, cells with this barcode from conditions treated with JNK-IN-8 appeared to be distributed across clusters and areas where these barcoded cells were not found in conditions without JNK-IN-8 treatment. In summary, twin clones largely maintained similar transcriptional fate types across conditions but showed increased transcriptional heterogeneity (more cells of the same barcode appearing in different clusters) when co-treated with JNK-IN-8 **(Supplemental Figure 3C)**. We then sampled 11 additional barcodes from the FateMap dataset. We present heatmaps of these 12 barcodes (BC1-BC12) in 12 separate blocks. The heatmaps reveal that for the majority of barcodes (8/12), the cell fate distribution in the low-to-high-dose switch condition closely resembles that of the low-dose conditions. For some barcodes (2/12), the low-to-high switch condition shows a distribution more similar to the high-dose condition. One barcode (1/12) doesn't completely match either the low-dose or high-dose patterns, while another (1/12) shows a similar distribution across all conditions **(Supplemental Figure 3D)**. Importantly, we

observed that co-treatment with JNK-IN-8 seemed to lead to more spread across clusters for each barcode lineage. These data suggest that cells largely maintain their transcriptional state when transitioning from low to high dose, but this persistence of cell fate can be altered by JNK-IN-8 treatment.

A crucial control for the JNK inhibitor experiments was to verify that treatment with JNK-IN-8 alone at the 1 μM dose does not inherently cause cell death or prevent cell growth in the WM989 melanoma system. We observed that cells treated with 14 days of JNK-IN-8 (1 μM) grew to be confluent in that time **(Supplemental Figure 3E)**. This result indicates that the duration and dose of JNK-IN-8 used in these experiments do not cause drastic cell death or prevent cell growth, showing that any observed effects are not due to JNK-IN-8 toxicity alone.

We also wondered how much adaptation is necessary for the initial transition from primed to resistant. We test this by co-treating naive cells with high-dose trametinib and 1 μM JNK-IN-8 for the first two weeks before we remove the JNK-IN-8 and allow cells to continue growing in 5 nM trametinib **(Supplemental Figure 3F)**. We observe a general decrease in the number of resistant colonies formed in this condition as compared to treatment with high-dose trametinib alone for the entire 4 weeks. This supports the notion that cellular adaptation is important for generally surviving this dose (i.e., simply being primed at the point of therapy initiation is not enough to become resistant if JNK is severely inhibited).

In addition to inhibiting AP-1 through JNK inhibition, we sought to replicate the results with another pharmacological inhibitor of AP-1, T-5224, a small molecule inhibitor that blocks the DNA binding activity of c-Fos/c-Jun **(Supplemental Figure 3G)**. In two biological replicates, we observed that co-treatment of 1μM T-5224 with low-dose trametinib (1 nM) for two weeks, followed by treatment with low-dose trametinib alone does not drastically alter the number of colonies that survived and became resistant at the end of the experiment compared to cells that only received low-dose trametinib for that duration. With this dose and treatment duration of T-5224, there was no notable effect on the survival of cells exposed to a high dose of trametinib. However, for cells that received co-treatment of 1 μM T-5224 with low-dose trametinib (1 nM) for two weeks, followed by treatment with high-dose trametinib (5 nM), there was a decrease in the number of final colonies. These data demonstrate that a second pharmacological inhibitor of AP-1 replicated the effects seen with JNK inhibition, further supporting the role of AP-1 in cellular adaptation to therapy.

Supplemental Figure 4

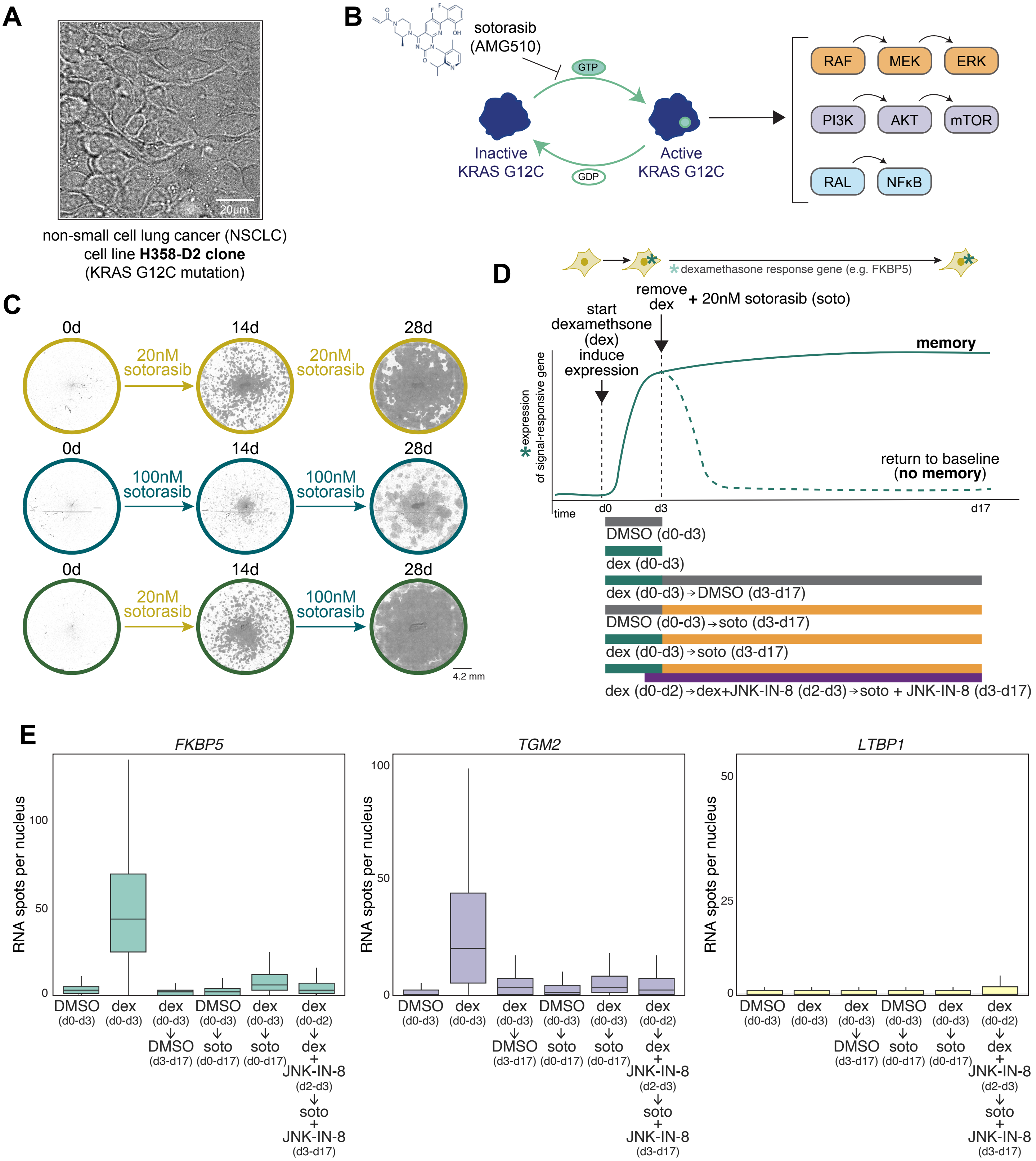

Supplemental Figure 4. Adaptation and memory also observed in H358 cell line of non-small cell lung cancer

(A) Phase-contrast image of single-cell clone (D2) of non-small cell lung cancer line H358 with a KRAS G12C mutation (H358-D2).
(B) Diagram showing the mechanism of action of sotorasib (AMG510) - covalently binding to inactive KRASG12C and preventing the GDP-GTP exchange that changes the mutant KRAS to the active conformation.
(C) Representative images of colonies at approximately day 0 (therapy initiation), day 14 (dose-switch), and day 28 (endpoint) for low dose alone, high dose alone, and low-to-high-dose switch conditions (n=1).
(D) Schematic of the hypothesis that the transition from primed to fully resistant cell induces the formation of cellular memories of the state of the cell at the time of therapy application (top); corresponding experimental design (bottom) for H358-D2 cells stimulated with dexamethasone then treated with 20 nM sotorasib.
(E) Boxplots of single-molecule RNA FISH quantification per nuclei of *FKBP5*, *TGM2*, and *LTBP1* expression under control and experimental conditions. A minimum of 237 cells were quantified in each condition. For each cell quantified, all genes are measured. All cells in each condition are from one biological replicate.

To test the generality of the concepts of memory described here, we conducted key experiments to look for evidence of the same adaptive behavior in an entirely different cell line **(Supplemental Figure 4A)** with a different drug and mechanism of action **(Supplemental Figure 4B)**. Here we used a bronchioalveolar carcinoma line (H358) and derived a single cell clone from the parental line that behaved similarly to the parent line to rule out the possibility of any genetic differences confounding potential observations of adaptation. We demonstrate that the same adaptive behavior with sotorasib, a KRAS G12C-specific inhibitor (low-to-high drug dose adaptation) **(Supplemental Figure 4C)** in which low dose alone for 4 weeks results in more resistant colonies than high dose alone for 4 weeks, and switching from low to high dose at the 2 week point results in as many resistant colonies as the low dose condition, demonstrating that cells are adapting while being exposed to the therapy at low dose (note that the number of resistant colonies was not quantified in this experiment since this cell line with this drug treatment results in resistant colonies that expand into each other, making accurate quantification difficult without consistent timelapse imaging). We also tested to see if dexamethasone-induced memory (see Fig. 3) occurs in this cell line as well and found that dexamethasone induced expression of *FKBP5* and *TGM2* were able to be carried as a passenger memory in this system (*LTBP1* did not show induction in this system even directly after dexamethasone treatment) **(Supplemental Figure 4D-E)**. The results in this cell line with a different drug and mechanism of action, along with supporting evidence from literature in other systems suggests that the concepts of memory demonstrated with the WM989 melanoma system may have more generalizable implications.

Supplemental Figure 5

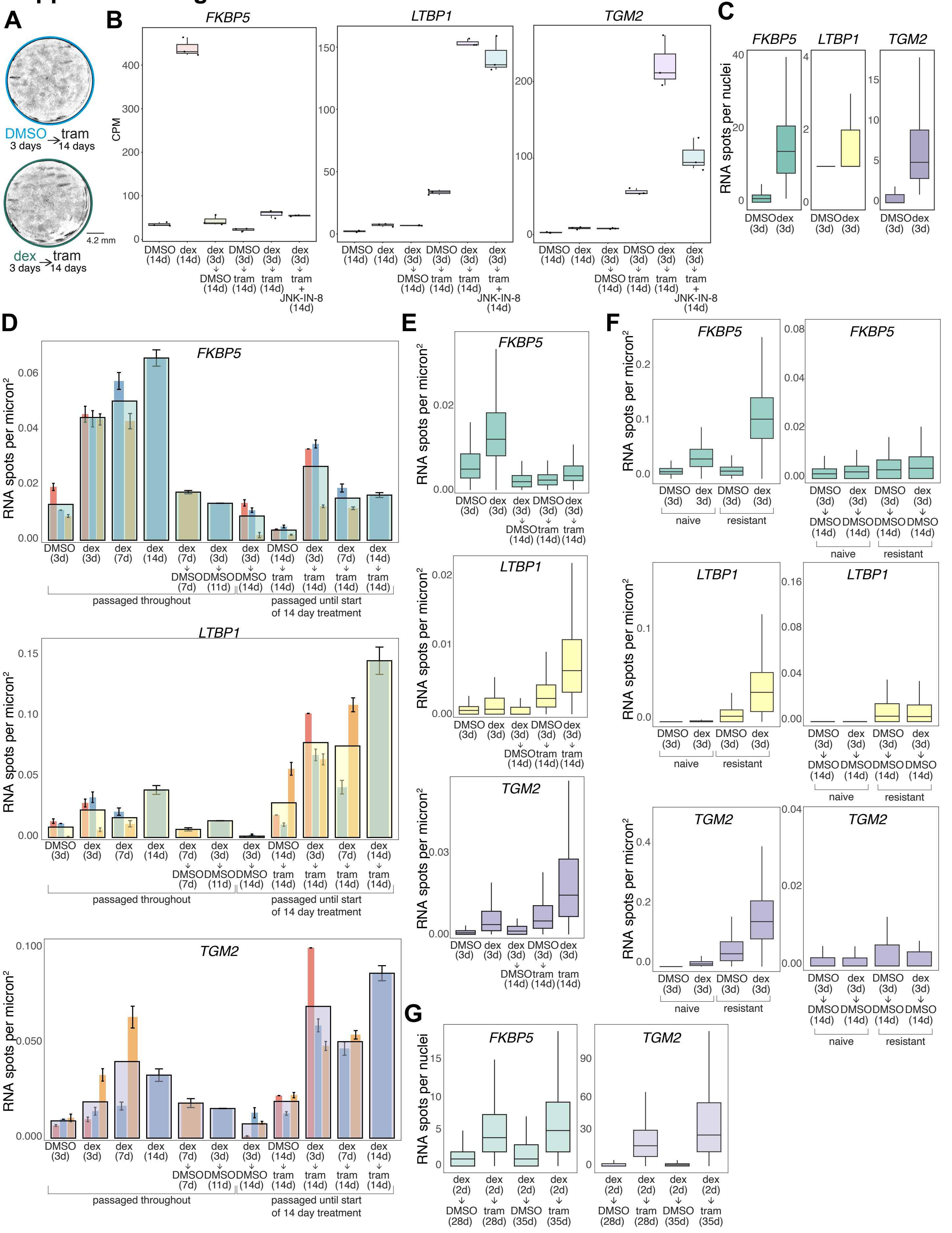

Supplemental Figure 5. Validation of dexamethasone-induction and reversion of gene expression in naive and resistant cells

(A) Phase-contrast images of WM989-A6-G3 cells grown in various conditions (n=1).
(B) Bulk RNA sequencing data for *FKBP5*, *LTBP1*, and *TGM2* under various conditions as shown.
(C) Boxplots showing gene expression (RNA counts per nucleus) in crowded cell conditions (n=1).
(D) Single-molecule RNA FISH quantification of *FKBP5*, *LTBP1*, and *TGM2* expression under various dexamethasone treatment durations. Data from 3 biological replicates (not all conditions present in each replicate). Larger overlaying bar plots represent the average of each condition. Smaller underlying bars represent individual replicate averages, consistently color-coded across conditions and genes. Error bars show standard error of the mean (SEM). The number of days spent in each condition are noted below each treatment.
(E) Single-molecule RNA FISH quantification of *FKBP5*, *LTBP1*, and *TGM2,* expression under various conditions in a different clonal population of WM989 cells (H2B-GFP WM989 A6-G3-A10) (n=1).
(F) Comparison of dexamethasone response and memory in naive and resistant WM989 cells. (left) boxplots comparing the induction of *FKBP5*, *LTBP1*, and *TGM2* expression in naive versus resistant cells in response to dexamethasone treatment. (right) Boxplots comparing the decrease in expression of *FKBP5*, *LTBP1*, and *TGM2* in naive versus resistant cells after dexamethasone removal (n=1).
(G) Boxplots showing showing gene expression (RNA counts per nucleus) for *FKBP5* and *TGM2* for naive WM989 cells exposed to dexamethasone for 2 days prior to either 28 or 35 days of DMSO or trametinib treatment (n=1).

To demonstrate that the transition from primed to fully resistant cells induces the formation of cellular memories, we tested whether we could cause the formation of a memory of a "passenger" gene's expression in response to dexamethasone as an inducer. We first tested to make sure that 3 days of dexamethasone treatment (the primary exposure duration we would use), would not change the ability of these cells to survive and form colonies if treated with 1 nM trametinib **(Supplemental Figure 5A)**. We observed no obvious changes in colony formation in 1 nM trametinib after 3 days of dexamethasone treatment compared to 3 days of DMSO treatment.

To identify potential evidence of memory formation, we conducted bulk RNA sequencing on WM989 cells under various treatment conditions, as shown **(Supplemental Figure 5B)**. We found a subset of genes that showed potential for learning, designed single-molecule RNA FISH probes for six of these genes, and proceeded with the first three probe sets to test our hypothesis (*FKBP5*, *LTBP1*, and *TGM2*). These genes displayed significantly higher induction when dexamethasone treatment was followed by trametinib, compared to trametinib treatment alone, and this enhanced expression was reduced when JNK-IN-8 was added, suggesting that learning occurs at loci responsive to trametinib and is partially dependent on AP-1 activity.

After seeing the results from the experiment in Figure 3B, we conducted a series of experiments to rule out possible confounding factors that may suggest alternative explanations to the memory formation we observed. Since we had observed in the single-molecule RNA FISH data collected in Figure 3B that conditions where cells were grown in dexamethasone for 3 days followed by DMSO for 14 days showed a global downregulation of gene expression, including in the expression of housekeeping gene hUBC, we induced target gene expression with 3 day dexamethasone treatment in high cell density conditions **(Supplemental Figure 5C)** to confirm that cells in high density situations (such as after continual growth in DMSO for weeks at a time) are still able to upregulate specific genes (*FKBP5*, *LTBP1*, *TGM2*). The observed induction of dexamethasone target genes at high cell density confirmed that the observed return to baseline expression in our main experiments for the dex -> DMSO conditions was not an artifact of high cell density but rather a true downregulation of gene expression.

Since bulk RNA sequencing and target gene selection were based on 14 days of dexamethasone exposure, while the validation by smFISH was based on 3 days of dexamethasone exposure, we measured the effects of a longer dexamethasone exposure on the expression of *FKBP5*, *LTBP1*, and *TGM2* **(Supplemental Figure 5D)**. We found a general trend of increased gene expression as the duration of dexamethasone exposure increased and a decrease in gene expression towards baseline when dexamethasone was removed, demonstrating that longer dexamethasone treatment generally led to higher gene induction. Importantly, in this experiment, cells were passaged every 3-4 days (n=3; not all conditions were represented in all replicates). In these conditions, gene expression increased during treatment and decreased upon dexamethasone removal despite cell passaging, demonstrating the reversibility of the effect and ruling out potential changes in expression resulting from prolonged culture without passaging. We also used this time course to test trametinib-induced memory to see the dynamics of memory with a longer dexamethasone exposure time while also passaging the cells prior to exposure to trametinib to prevent overcrowding. We observed a general trend where we observed a stronger trametinib-induced memory effect for *FKBP5*, *LTBP1*, and *TGM2* by single-molecule RNA FISH. Importantly, these results demonstrate that passaging cells during the experiment does not alter the memory formation and retention effects we have observed. This control supports the robustness of our findings across different experimental conditions and timescales, reinforcing the validity of our cellular memory model in the context of drug resistance acquisition.

We also performed the dexamethasone-induced passenger memory experiment in a different clonal cell line of WM989s to further validate our finding from Figure 3B **(Supplemental Figure 5E)**. The results here closely resemble the results seen within Figure 3B, suggesting that the findings from Figure 3B are not an artifact of that single clonal population. (Note that JNK inhibition was not tested in this experiment with the H2B-GFP WM989 A6-G3-A10 cells.

To eliminate the possibility that resistant cells have different induction and reversion timescales compared to therapy-naive cells, we conducted an experiment using a population of resistant cells that had never been exposed to dexamethasone. Resistant cells (and, as a control, naive cells) were treated with dexamethasone for 3 days, followed by 11 days of culture without dexamethasone **(Supplemental Figure 5F)**. We observed that the expression of our target genes (*FKBP5*, *TGM2*, and *LTBP1*) increased upon dexamethasone induction and reverted to baseline levels after dexamethasone removal. This control experiment (n=1) showed that resistant cells do not have an inherently altered regulatory system for these genes, as they exhibit similar patterns of gene induction and reversion to baseline as naive cells. In line with our other experimental observations, this evidence points to a critical time window for memory formation that occurs early in the trametinib treatment process.

Lastly, to address the question of the permanence of the memory of dexamethasone exposure, we exposed naive WM989 cells to dexamethasone for 2 days before replacing the dexamethasone with either DMSO (vehicle control) or 1 nM trametinib (memory condition) **(Supplemental Figure 5G)**. We find that at 28 days and at 35 days after dexamethasone exposure, cells still retain a memory of *FKBP5* and *TGM2* induction as a result of trametinib treatment compared to DMSO control. The 28-day and 35-day experiments shown here are from two separate biological replicates.

Supplemental Figure 6

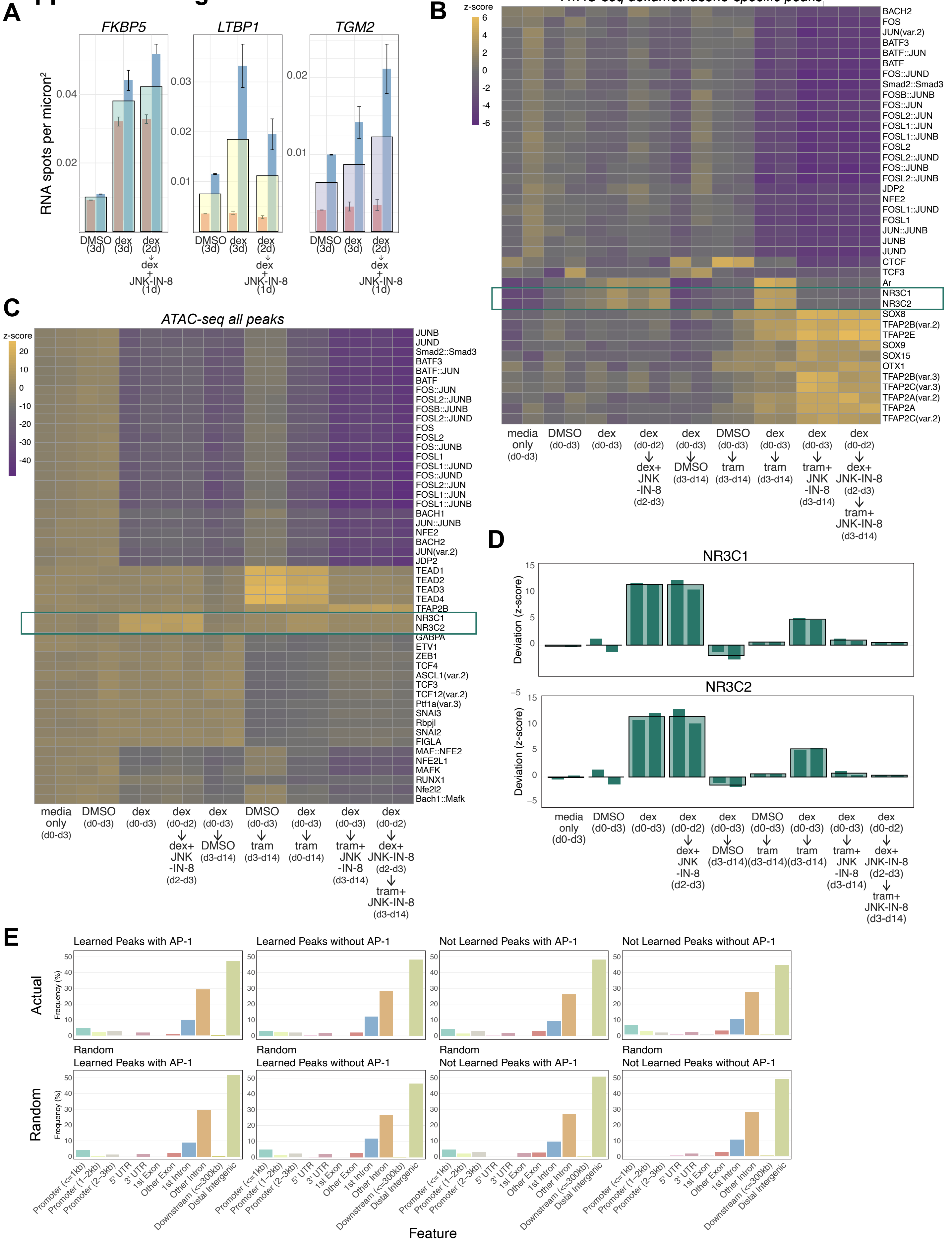

Supplemental Figure 6. Validation of AP-1 inhibition by JNK inhibition and comprehensive chromatin accessibility analysis supports an AP-1-dependent mechanism of memory formation.

(A) Bar plots showing results from two biological replicates of the effects of dexamethasone treatment for two days, followed by co-treatment of JNK-IN-8 (1μM) and dexamethasone for one day. Larger overlaying bars represent the average of each condition. Smaller underlying bars show individual replicate averages, consistently color-coded across conditions and genes. Error bars indicate SEM.
(B) chromVAR heatmap showing changes in transcription factor signatures in all experimental conditions for peaks with increased chromatin accessibility after 3 days of dexamethasone treatment, compared to 3 days of DMSO, and peaks that were not induced by trametinib treatment alone. (A subset of this heatmap is shown in Figure 3D).
(C) chromVAR heatmap demonstrating changes in Glucocorticoid receptor and AP-1 transcription factor signatures in experimental conditions for all variable peaks.
(D) Bar graphs of chromVAR deviation z-scores demonstrating changes in transcription factor signatures for NR3C1(top) and NR3C2 (bottom) for all conditions performed in the experiment. Large bars represent the mean for each condition, and smaller underlying bars show the two biological replicates. A subset of this data is shown in Figure 3D.
(E) Barplots showing frequency of genomic features of learned vs. not learned ATAC-seq peaks with and without AP-1 (top) compared to a random bootstrapped subset of peaks matched to the sample size of each corresponding test set (bottom). All ATAC sequencing data shown in this figure are from one biological replicate.

In Figure 3B, we showed the effects of inhibiting AP-1 with JNK-IN-8 overlapping with the last day of dexamethasone induction. To rule out the possibility that JNK-IN-8 treatment on the final day of a three-day dexamethasone exposure affected the expression of our target genes, we measured expression of *FKBP5*, *LTBP1*, and *TGM2* by single-molecule RNA FISH at the end of the three-day treatment **(Supplemental Figure 6A)**. We found that JNK-IN-8 treatment on the third day of a three-day dexamethasone exposure did not significantly alter the expression of these genes as compared to dexamethasone treatment alone for 3 days, suggesting that the decreased gene expression observed when JNK-IN-8 and trametinib are co-administered in our main experiments is likely due to the inhibition of AP-1 during the memory formation process, rather than an immediate effect of JNK-IN-8 on dexamethasone-induced gene expression.

To further analyze changes in chromatin accessibility associated with memory formation, we generated a subset of peaks to analyze that showed both an increase in chromatin accessibility in the 3 days of dexamethasone conditions (3 days of dexamethasone conditions compared to 3 days of DMSO conditions) but also no increase simply as a result of treatment with trametinib (3 day of DMSO treatment followed by 11 days of trametinib treatment compared to 3 days of DMSO). This subset of peaks allowed us to focus specifically on the effects of dexamethasone-responsive peaks to the exclusion of the effects of trametinib treatment. In this subset, we saw that accessibility increased in regions with the GR element upon dexamethasone treatment and not with trametinib alone, but when dexamethasone was removed and trametinib was added (the memory formation condition), we observed not only maintenance but actually an increase in chromatin accessibility of regions that contain sequences with GR signatures **(Supplemental Figure 6B)**. We observed similar effects when examining all peaks as we had when comparing peaks specifically responsive to dexamethasone and excluding trametinib-induced peaks **(Supplemental Figure 6C)**. In short, whether we look at all variable peaks or the subset of peaks that are responsible to dexamethasone but not induced by trametinib alone, we see memory of dexamethasone-induced increased chromatin accessibility at GR binding site that is induced by trametinib and blocked by JNK-inhibitor, regardless of whether the JNK inhibition started on the last day of dexamethasone treatment or at the same time as the trametinib treatment. Collectively these results collectively support our findings that AP-1 plays a crucial role in maintaining the chromatin accessibility changes associated with memory formation.

We also extended our analysis to include all experimental conditions as shown in Figure 3D. Bar graphs of chromVAR deviation z-scores demonstrating changes in transcription factor signatures for NR3C1 (top) and NR3C2 (bottom) are presented for all conditions performed in the experiment **(Supplemental Figure 6D)**. Large bars represent the mean for each condition, while smaller underlying bars show the two replicates. This comprehensive analysis revealed the same pattern of changes observed in the heatmaps; specifically, we observed increased accessibility at GR binding sites with dexamethasone treatment, which was maintained or even enhanced when followed by trametinib treatment. Importantly, this maintenance of accessibility was blocked by JNK inhibition, regardless of whether JNK inhibition started on the last day of dexamethasone treatment or concurrently with trametinib treatment. These results further support our conclusion that AP-1 activity is crucial for maintaining the chromatin accessibility changes associated with memory formation.

To answer the question of whether adaptation would occur mostly at specifically annotated genomic features or chromosomal domains (e.g. transcriptional start sites, 5' or 3' UTRs, proximal or distal promoters, exonic or intronic regions, intergenic regions), we subsetted learned peaks that had an AP-1 binding site within 500 basepairs of the center the peak, learned peaks without an AP-1 binding site, not learned peaks with an AP-1 binding site, and not learned peaks without an AP-1 binding site - comparing the frequency of peaks across these conditions to a random subset of peaks (random subsampling of the peaks increased specifically in the Dex condition that matched the sample size of each corresponding test set) **(Supplemental Figure 6E)**, we find no drastic differences of adaption happening at any specific genomic features or domains - however, given the timescale of experiment and the number of learned and not learned peaks in our data, it is possible that there may be specific domains or features more commonly associated with memory formation that we are too underpowered to be able to deduce here.

Supplemental Figure 7

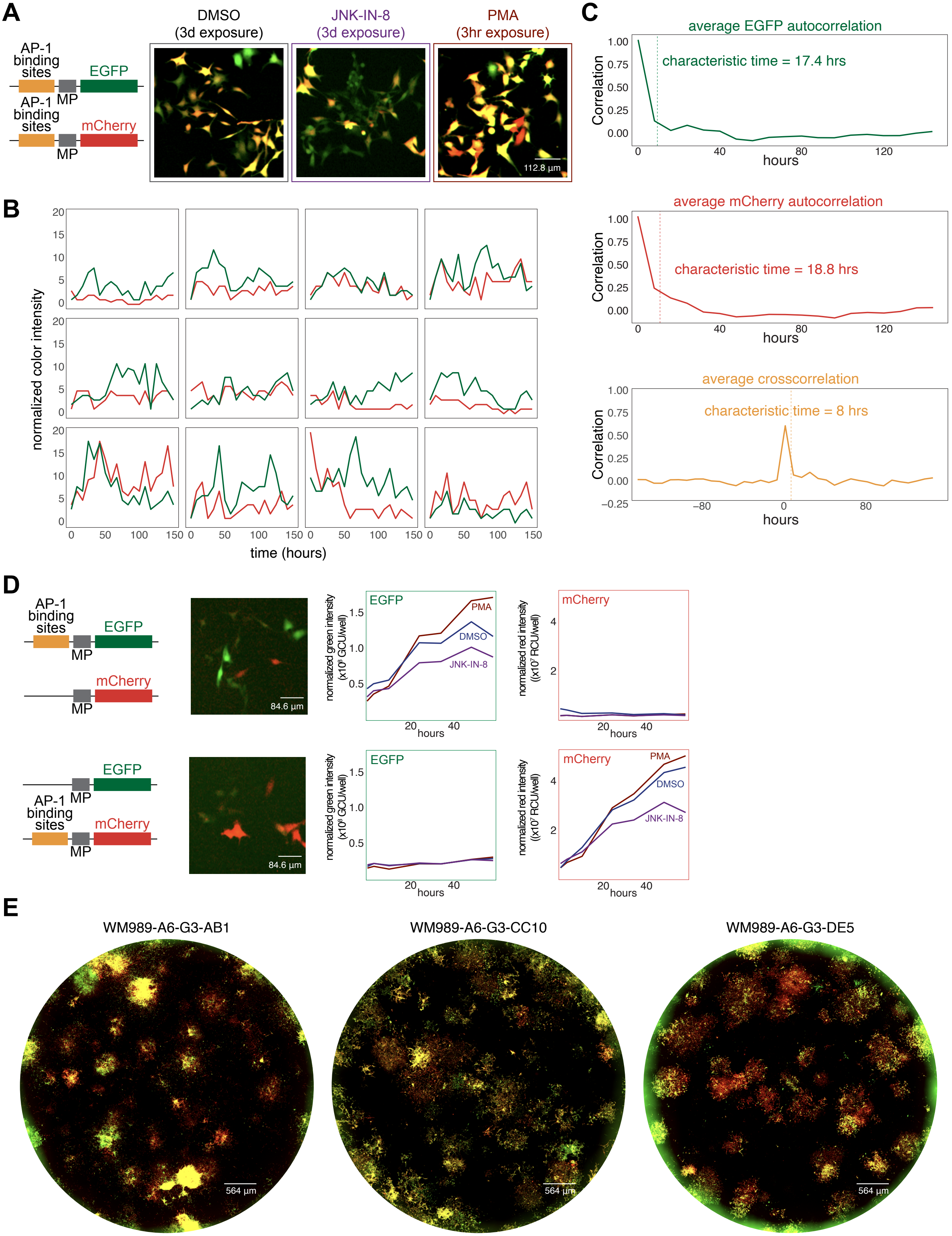

Supplemental Figure 7. Validation of the dynamics, stability, and specificity of AP-1 reporter cells
(A) Sample images of a single-cell cloned population of WM989 A6-G3 cells transduced with the two AP-1 reporter constructs (WM989 A6-G3-BE5) (left). The right panel shows images collected at 7 days after either a 3-day exposure of DMSO, a 3-day exposure of 1µM JNK-IN-8, or a 3-hour exposure of PMA. All three images are contrasted the same.
(B) Twelve independent cell traces of EGFP and mCherry fluorescence intensities. Each graph represents a single WM989A6-G3-BE5 AP-1 reporter cell tracked over 144 hours. Color intensity is calculated as the mean fluorescence intensity of the cell minus the median of the fluorescence intensity of a 10-pixel annulus around that cell. These cell traces show that there are various amplitudes of fluctuations in EGFP and in mCherry.
(C) Autocorrelation and cross-correlation analysis for EGFP and mCherry signals (n=34 cells). EGFP autocorrelation shows an average characteristic time of 17.4 hours, while mCherry autocorrelation has an average characteristic time of 18.8 hours. The EGFP-mCherry cross-correlation displays an average correlation time of 8 hours. Characteristic time is calculated as when the correlation drops below a threshold of 1/e of its initial value.
(D) Top: normal AP1-EGFP reporter construct and an mCherry construct co-transduced with only the minimal promoter and no AP-1 binding site. Bottom: normal AP1-mCherry reporter construct co-transduced with an EGFP construct with only the minimal promoter and no AP-1 binding site. Clonal populations of each combination were isolated by dilution cloning. To the right of the schematic are day 1 images of a clonal population of cells containing each of the AP-1 driven and non-AP-1 driven constructs. Graphs to the right show Incucyte S3 well measurements of normalized green and red fluorescence intensity values for that clonal population of cells across time under DMSO, PMA, or JNK-IN-8 treatment (n=1).
(E) Whole well images of colonies formed after 28 days of treatment with 1 nM trametinib for 3 different clonal populations of the WM989-A6-G3 reporter cells: (left) WM989-A6-G3-AB1, (middle) WM989-A6-G3-CC10, (right) WM989-A6-G3-DE5.

As a first test of the AP-1 dual reporter system, we confirmed that a clonal population of WM989-A6-G3-BE5 reporter cells showed a notable reduction in fluorescence intensity resulting from treatment with JNK-IN-8 and an overall increase in fluorescence for cells treated with a short-term exposure of PMA, demonstrating the responsiveness of the reporter system to AP-1 inhibition and activation, respectively **(Supplemental Figure 7A)**. We then sought to characterize the baseline fluctuations of fluorescent reporters in therapy-naive cells. **Supplemental Figure 7B** shows twelve independent cell traces of EGFP and mCherry fluorescence intensities for a WM989-A6-G3-BE5 AP-1 reporter cell tracked over 144 hours. These cell traces showed that there were various amplitudes of fluctuations in EGFP and mCherry expression levels. Importantly, we observed instances when the mCherry and EGFP intensities change in the same direction (increased or decreased) from one time point to another and instances when the mCherry and EGFP change in opposite directions from one time point to another. To quantify these fluctuations at baseline, we calculated autocorrelation and cross-correlation for EGFP and mCherry signals (n=34 cells). EGFP autocorrelation showed an average characteristic time of 17.4 hours, while mCherry autocorrelation had an average characteristic time of 18.8 hours. The EGFP-mCherry cross-correlation displayed an average cross-correlation time of 8 hours. Here characteristic time is calculated as when the correlation drops below a threshold of 1/e of its initial value. These values of characteristic times for EGFP and mCherry autocorrelation and crosscorrelation indicate that expression levels of EGFP and mCherry change roughly daily and that EGFP and mCherry expression do not sustain correlation for long periods **(Supplemental Figure 7C)**.

We also sought to verify that the reporter system was specifically dependent on the AP-1 binding sites. To achieve this, we created an internally controlled system where we either co-transduced into WM989 A6-G3 cells the normal AP1-EGFP reporter construct and an mCherry construct with only the minimal promoter and no AP-1 binding sites, or we co-transduced the normal AP1-mCherry reporter construct with an EGFP construct with only the minimal promoter and no AP-1 binding sites **(Supplemental Figure 7D)**. These images

confirm that there is very low but present expression of the fluorescent protein without the AP-1 binding sites. Graphs below show that within the same clonal population, the AP-1 responsive changes in fluorescence were only seen for the fluorescent protein that has the AP-1 binding site upstream, confirming the specificity of the reporter system to AP-1 activity **(Supplemental Figure 7D)**.

To demonstrate that the phenomenon we observed in Figure 4C was not only a result of the exact site of integration of the reporter constructs in the BE5 clone, we isolated and generated trametinib-resistant colonies with 3 additional reporter cell lines, all dilution-cloned and expanded from a single cell **(Supplemental Figure 7E)**. For reporter lines AB1, CC10, and DE5, we observed diverse colors of resistant colonies in all three additional cell lines, as we saw with the BE5 line, suggesting that the memory formation process is a general phenomenon independent of the specific integration pattern of the reporter constructs.

Supplemental Figure 8

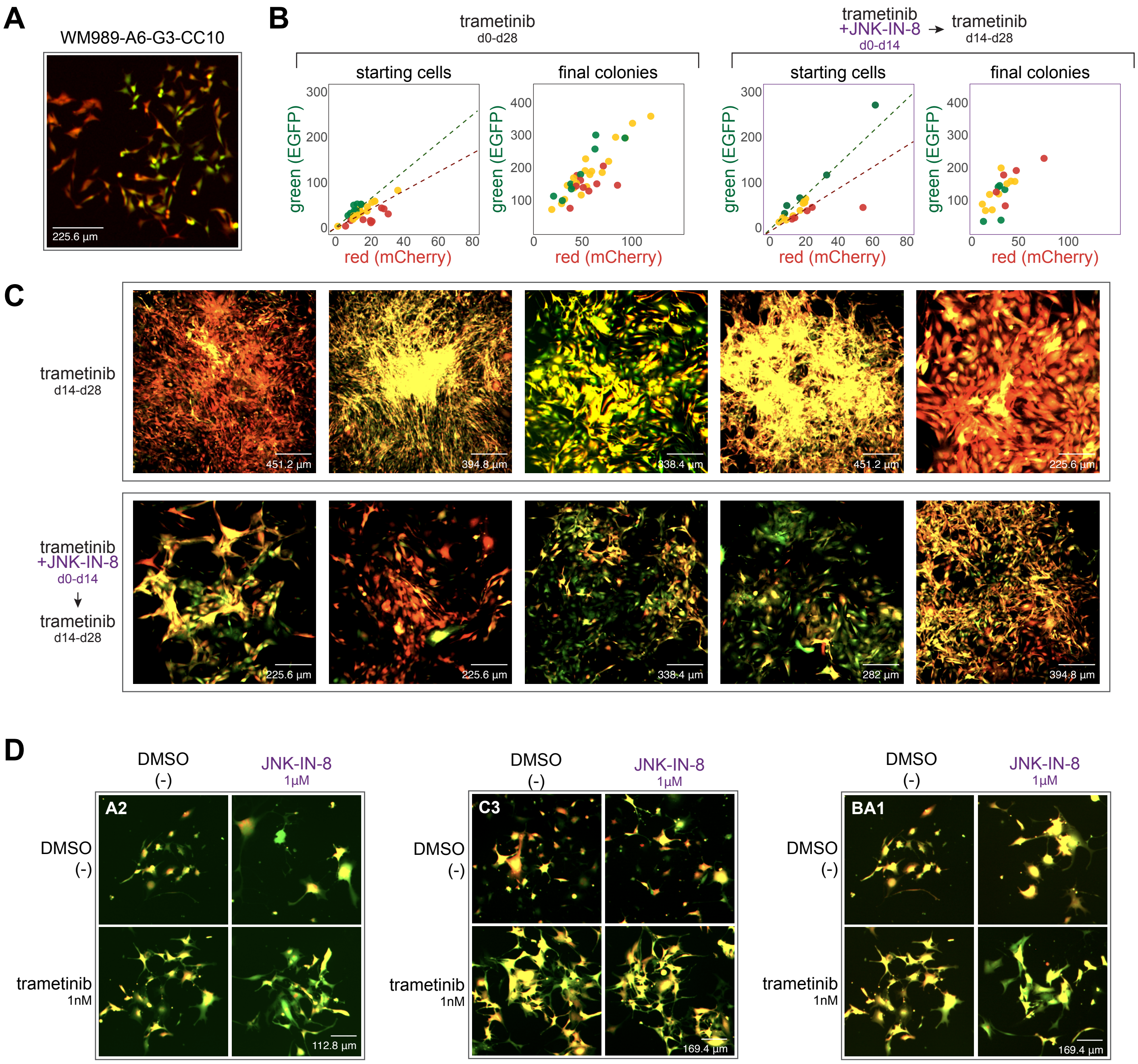

Supplemental Figure 8. Validation of the dynamics of a second clone of AP-1 reporter cells - WM989-A6-G3-CC10

(A) Image showing cell-to-cell variability in expression in a single-cell-derived clone of WM989 A6-G3 cells transduced with both reporter constructs (WM989-A6-G3-CC10)

(B) Scatter plots of fluorescence quantifications from Incucyte S3 images of WM989-A6-G3-CC10 cells grown under two conditions: Left: 1 nM trametinib alone for 28 days. Right: 1 nM trametinib and 1 μM JNK-IN-8 co-treatment for days 0-14, followed by trametinib alone for days 14-28. Colonies were traced back through time-lapse images to identify their originating cells. The initial red-to-green fluorescence intensity ratio of these starting cells was used to categorize the initial 'color' of the cell: top 25th percentile as red, bottom 25th percentile as green, and the remainder as yellow. This initial categorization is used to determine the color coding of the final colonies. The same system is applied for the JNK-IN-8 panel, with the color coding of the final colonies determined according to the starting cell red-to-green ratio for cells in the JNK-IN-8 treatment condition (n=1).

(C) Day 28 images of the colonies shown in panel A. The top panel shows trametinib alone for the duration of the experiment, while the bottom panel shows trametinib and JNK-IN-8 co-treatment during the first 14 days and trametinib alone for the next 14 days. All images are contrasted the same for each channel and are from the same biological replicate.

(D) Images of three manually isolated resistant colony clones A2, BA1, and C3 cultured for more than 1 month after initial colony formation and isolation. Cells were plated at day 0 and either continued to be grown in trametinib or had trametinib removed (DMSO alone), and for both those conditions, with and without 1 μM JNK-IN-8. These images were collected after 7 days of this treatment (n=1).

To validate our findings from Figure 4 in another clonal cell line, we first demonstrate the baseline variability of the CC10 clone **Supplemental Figure 8A**. We also performed time-lapse tracking of cells through the process of colony formation in the CC10 clone. We quantified the fluorescence levels of the initial state of the cell and then connected those cells to the ultimate therapy-resistant colony and found that cells that were initially more mCherry or EGFP-positive ended up forming resistant colonies that largely preserved those differences **(Supplemental Figure 8B)**, as we saw for the BE5 clone (Fig. 4E). JNK inhibition led to a reduction in the induction of the levels of both reporters as well as a more noticeable variation of colors within colonies, again suggesting that blocking memory formation allows for cell fates to be more malleable **(Supplemental Figure 8B,C)**. To visualize the durability of therapy resistance memory and its dependence on AP-1, we isolated five trametinib-resistant colonies grown from WM989 A6-G3-CC10 and grew three of these individual therapy-resistant colonies (A2, BA1, C3) with and without trametinib and with and without JNK inhibitor for another 7 days. We found that resistant colonies maintained high expression of both fluorescent proteins after a month in trametinib. This signal persisted largely unchanged when grown with or without trametinib and JNK inhibitor for an additional week, suggesting that once established, the epigenetic memory of resistance is maintained by a mechanism independent of trametinib effects or AP-1 activity **(Supplemental Figure 8D).**

Supplemental Figure 9

A

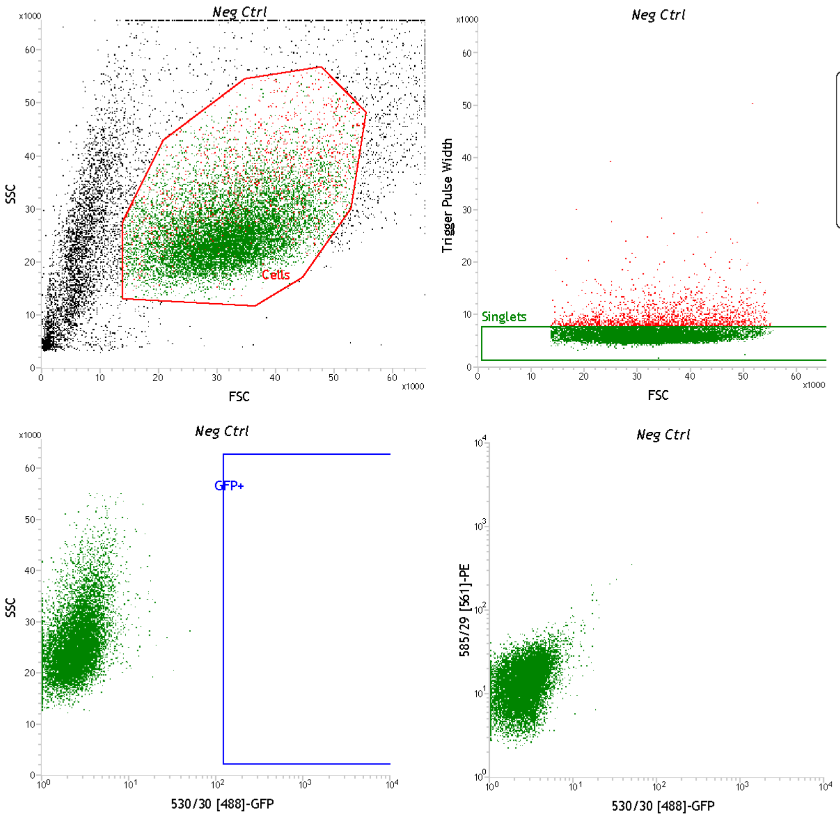

Populations: *Neg Ctrl*

Populations	Events	% Total	% Parent
All Events	20,000	100.00%	####
Cells	14,036	70.18%	70.18%
Singlets	12,254	61.27%	87.30%
GFP+	0	0.00%	0.00%

B

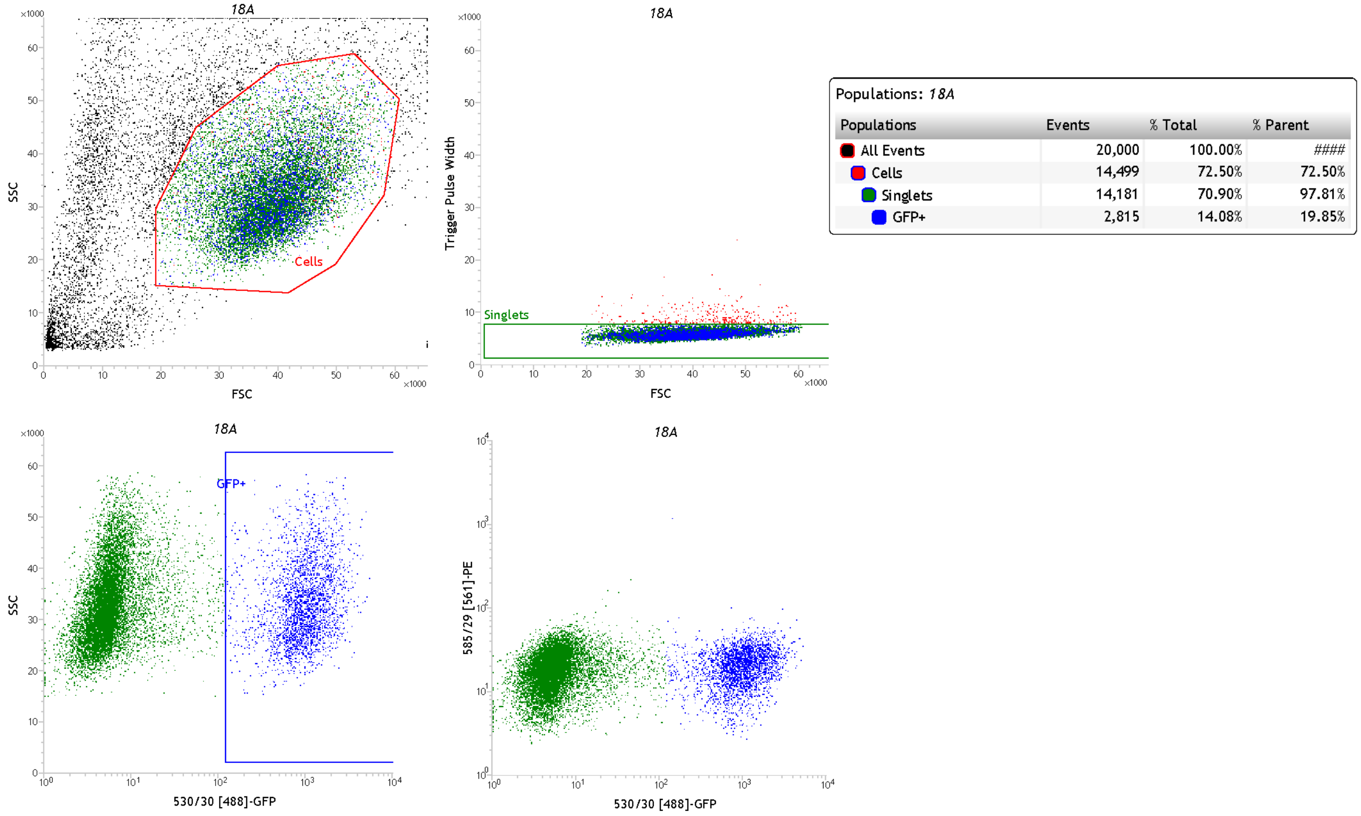

Populations: *18A*

Populations	Events	% Total	% Parent
All Events	20,000	100.00%	####
Cells	14,499	72.50%	72.50%
Singlets	14,181	70.90%	97.81%
GFP+	2,815	14.08%	19.85%

Supplemental Figure 9. Gating strategy for barcoded (GFP+) cell lines used in FateMap experiments
(A) Flow cytometry plots and gating strategy for WM989 A6-G3 melanoma cells (example of negative control)
(B) Flow cytometry plots and gating strategy for WM989 A6-G3 melanoma cells (example of cells transduced for FateMap experiments)

Supplementary Tables

Supplementary Table 1: Cloning primers for generation of AP-1 responsive fluorescent reporters

primer name	sequence
A	attgtcgacctacttgtacagctcgt
B	gcgaccggtaagcttcaagatgaggcaa
C	atcgatcgatggatcctgagtcagtgactc
D	atcgatcgatgaattcagcttgaataaaat

Supplementary Table 2: Primers used to generate gDNA barcode sequencing libraries

primer name	sequence	index	stagger
LentiEFS_GFP_NGS_i5.2.1	AATGATACGGCGACCACCGAGATCTACACTAGATCGCACACTCTTTCCCTACACGACGCTCTTCCGATCTNHNNNNTCGACTAAACGCGCTACTTG	TAGATCGC	None
LentiEFS_GFP_NGS_i5.2.2	AATGATACGGCGACCACCGAGATCTACACCTCTCTATACACTCTTTCCCTACACGACGCTCTTCCGATCTNHNNNNCGTCGACTAAACGCGCTACTTG	CTCTCTAT	CG
LentiEFS_GFP_NGS_i5.2.3	AATGATACGGCGACCACCGAGATCTACACTATCCTCTACACTCTTTCCCTACACGACGCTCTTCCGATCTNHNNNNGACTCGACTAAACGCGCTACTTG	TATCCTCT	GAC
LentiEFS_GFP_NGS_i5.2.4	AATGATACGGCGACCACCGAGATCTACACAGAGTAGAACACTCTTTCCCTACACGACGCTCTTCCGATCTNHNNNNATAGTCTCGACTAAACGCGCTACTTG	AGAGTAGA	ATAGTC
LentiEFS_GFP_NGS_i5.2.5	AATGATACGGCGACCACCGAGATCTACACGTAAGCAGACACTCTTTCCCTACACGACGCTCTTCCGATCTNHNNNNTACGTCGACTAAACGCGCTACTTG	GTAAGCAG	TACG
LentiEFS_GFP_NGS_i5.2.6	AATGATACGGCGACCACCGAGATCTACACACTGCGTAACACTCTTTCCCTACACGACGCTCTTCCGATCTNHNNNNCCTAATCGACTAAACGCGCTACTTG	ACTGCGTA	CCTAA
LentiEFS_GFP_NGS_i7.2.1	CAAGCAGAAGACGGCATACGAGATTAAGGCGAGTGACTGGAGTTCAGACGTGTGCTCTTCCGATCTGTCCTGCTGGAGTTCGTGAC	TAAGGCGA	None
LentiEFS_GFP_NGS_i7.2.2	CAAGCAGAAGACGGCATACGAGATCGTACTAGGTGACTGGAGTTCAGACGTGTGCTCTTCCGATCTAGGTCCTGCTGGAGTTCGTGAC	CGTACTAG	AG
LentiEFS_GFP_NGS_i7.2.3	CAAGCAGAAGACGGCATACGAGATAGGCAGAAGTGACTGGAGTTCAGACGTGTGCTCTTCCGATCTTCAAGTCCTGCTGGAGTTCGTGAC	AGGCAGAA	TCAA
LentiEFS_GFP_NGS_i7.2.4	CAAGCAGAAGACGGCATACGAGATTCCTGAGCGTGACTGGAGTTCAGACGTGTGCTCTTCCGATCTCACTTAAGTCCTGCTGGAGTTCGTGAC	TCCTGAGC	CACTTAA
LentiEFS_GFP_NGS_i7.2.5	CAAGCAGAAGACGGCATACGAGATCGACTCCTGTGACTGGAGTTCAGACGTGTGCTCTTCCGATCTTGTCCTGCTGGAGTTCGTGAC	CGACTCCT	T
LentiEFS_GFP_NGS_i7.2.6	CAAGCAGAAGACGGCATACGAGATTAGGCATGGTGACTGGAGTTCAGACGTGTGCTCTTCCGATCTCTTGAGTCCTGCTGGAGTTCGTGAC	TAGGCATG	CTTGA

Supplementary Table 3: Sequences of the smFISH probes used in this study

gene	sequence	probe
FKBP5	cctgtctttttggaggtaa	FKBP5_exonContinuous_1
FKBP5	cccactcttttgacaatctt	FKBP5_exonContinuous_2
FKBP5	actttgtctccaatcatcgg	FKBP5_exonContinuous_3
FKBP5	tccatttgacaattttcctt	FKBP5_exonContinuous_4
FKBP5	gcctttgccaagactaaaga	FKBP5_exonContinuous_5
FKBP5	catatctctcctttcttcat	FKBP5_exonContinuous_6
FKBP5	catattctggtttgcacagt	FKBP5_exonContinuous_7
FKBP5	gaattttagggagactgcca	FKBP5_exonContinuous_8
FKBP5	cctttgaaatcaaggagctc	FKBP5_exonContinuous_9
FKBP5	gtttggttctccggataatg	FKBP5_exonContinuous_10
FKBP5	tggatttgaatatccctctc	FKBP5_exonContinuous_11
FKBP5	caggtggatttctactgttg	FKBP5_exonContinuous_12
FKBP5	attggaatgtcgtggtcttc	FKBP5_exonContinuous_13
FKBP5	ctccagagctttgtcaattc	FKBP5_exonContinuous_14
FKBP5	aaccatatcttggtccaaga	FKBP5_exonContinuous_15
FKBP5	ctcagcattaggttcaatgc	FKBP5_exonContinuous_16
FKBP5	attctttggccttttcgaag	FKBP5_exonContinuous_17
FKBP5	gctccaatttttctttggta	FKBP5_exonContinuous_18
FKBP5	ttctctttgacaatggcagc	FKBP5_exonContinuous_19
FKBP5	tcccttgaagtatacggttc	FKBP5_exonContinuous_20
FKBP5	atcttcccatactgaatcac	FKBP5_exonContinuous_21
FKBP5	tattccatctctaaccagga	FKBP5_exonContinuous_22
FKBP5	cagaagctttcgattccttt	FKBP5_exonContinuous_23
FKBP5	cacatggccaggttcagaaa	FKBP5_exonContinuous_24
FKBP5	ggtgtattctctaagcttca	FKBP5_exonContinuous_25
FKBP5	ttgtcacagcattcaacagc	FKBP5_exonContinuous_26
FKBP5	atacaagcctttctcattgg	FKBP5_exonContinuous_27
FKBP5	ttggctgactcaaactcgtt	FKBP5_exonContinuous_28
FKBP5	ttttctggcacatggagatc	FKBP5_exonContinuous_29
FKBP5	gaacatgttggcgtatatcc	FKBP5_exonContinuous_30
FKBP5	ttccttttcattagtgacc	FKBP5_exonContinuous_31
FKBP5	aggtttctcttcttccattg	FKBP5_exonContinuous_32
LTBP1	tgtctgccttcttagatgaa	LTBP1_exonContinuous_1
LTBP1	tgagaatgtatctgctgggg	LTBP1_exonContinuous_2
LTBP1	aatcaccacactctgggaag	LTBP1_exonContinuous_3
LTBP1	aaagtacttgggcttgagca	LTBP1_exonContinuous_4

LTBP1	ggccatcaattcttgaaacc	LTBP1_exonContinuous_5
LTBP1	tgagcttcttttgtcttctg	LTBP1_exonContinuous_6
LTBP1	ataagatggtttcttggggc	LTBP1_exonContinuous_7
LTBP1	tacaccttgtagctgacatt	LTBP1_exonContinuous_8
LTBP1	cccatggtattcaaacactc	LTBP1_exonContinuous_9
LTBP1	tttgcaggtacatcgatagc	LTBP1_exonContinuous_10
LTBP1	tgaacagacagagggtgcat	LTBP1_exonContinuous_11
LTBP1	tcaggagctacttcaacagg	LTBP1_exonContinuous_12
LTBP1	cactggtaggttaatgcagt	LTBP1_exonContinuous_13
LTBP1	tcacagtattcacaccggaa	LTBP1_exonContinuous_14
LTBP1	tggacaagtgcttggattca	LTBP1_exonContinuous_15
LTBP1	ggcattcatcaatgtcttca	LTBP1_exonContinuous_16
LTBP1	gatatcttcacagtggtctc	LTBP1_exonContinuous_17
LTBP1	aaacactcttgtcctccaag	LTBP1_exonContinuous_18
LTBP1	cagtattaatgcagtctcct	LTBP1_exonContinuous_19
LTBP1	tgtcacaaaacccgtgactg	LTBP1_exonContinuous_20
LTBP1	ataacagaggcagcggaagg	LTBP1_exonContinuous_21
LTBP1	gttcacattcattcacatcc	LTBP1_exonContinuous_22
LTBP1	attatcacagagactggcgt	LTBP1_exonContinuous_23
LTBP1	aggggaagatttcgcagtta	LTBP1_exonContinuous_24
LTBP1	cttgcttacagtagcattca	LTBP1_exonContinuous_25
LTBP1	aactactggggtcttgacat	LTBP1_exonContinuous_26
LTBP1	agcatagtcatctgaatcct	LTBP1_exonContinuous_27
LTBP1	accattttcacatccattga	LTBP1_exonContinuous_28
LTBP1	tacatcgacacaggtcatct	LTBP1_exonContinuous_29
LTBP1	gttgttcaactcatcgcatt	LTBP1_exonContinuous_30
LTBP1	gtgttaatgcacttggcatt	LTBP1_exonContinuous_31
LTBP1	aggtcactgtctttctctaa	LTBP1_exonContinuous_32
TGM2	agatcacacctctctaagac	TGM2_exonContinuous_1
TGM2	cactgaaggtgagactgtct	TGM2_exonContinuous_2
TGM2	ttagtggaaaacgggccttg	TGM2_exonContinuous_3
TGM2	gagtccaggtacacagcatc	TGM2_exonContinuous_4
TGM2	tggtagataaagccctgctg	TGM2_exonContinuous_5
TGM2	cttgatgaacttggccgagc	TGM2_exonContinuous_6
TGM2	gcccaaaattccaaggtatg	TGM2_exonContinuous_7
TGM2	tctaggatcccatcttcaaa	TGM2_exonContinuous_8
TGM2	catcgttgcagttgaccatg	TGM2_exonContinuous_9
TGM2	cagcactggccatacttgac	TGM2_exonContinuous_10
TGM2	gagttgtagttggtcacgac	TGM2_exonContinuous_11

TGM2	gatgagaaggttgctgttct	TGM2_exonContinuous_12
TGM2	ccaaactcattgcggaagta	TGM2_exonContinuous_13
TGM2	agcagtggaagttccagatc	TGM2_exonContinuous_14
TGM2	atggcacgaactggaactgg	TGM2_exonContinuous_15
TGM2	catcgtacttggtgctcagg	TGM2_exonContinuous_16
TGM2	attgacctccgcaaagacaa	TGM2_exonContinuous_17
TGM2	gatttgtgcacagacccatc	TGM2_exonContinuous_18
TGM2	caacgatcagggaacggttg	TGM2_exonContinuous_19
TGM2	ctcttagtgctgatcttcag	TGM2_exonContinuous_20
TGM2	ttgtaggtgtgggtgatatc	TGM2_exonContinuous_21
TGM2	ccagtttgttcaggtggttc	TGM2_exonContinuous_22
TGM2	tgttggtgatgtgggcaaag	TGM2_exonContinuous_23
TGM2	aagatcccattgtagctgac	TGM2_exonContinuous_24
TGM2	tgaggttgagcaggtacttg	TGM2_exonContinuous_25
TGM2	ttctcagagaaaggctccag	TGM2_exonContinuous_26
TGM2	gaggatgcaaagaggaacgc	TGM2_exonContinuous_27
TGM2	taaggcagtcacggtatttc	TGM2_exonContinuous_28
TGM2	accttgatgaggttggactc	TGM2_exonContinuous_29
TGM2	ataactggctccacgaggag	TGM2_exonContinuous_30
TGM2	caggtccattctcaccttaa	TGM2_exonContinuous_31
TGM2	ccaatgatgacattccggaa	TGM2_exonContinuous_32
hUBC	atggtcttaccagtcagagt	hUBC_1
hUBC	gacattctcgatggtgtcac	hUBC_2
hUBC	gggatgccttccttatcttg	hUBC_3
hUBC	atcttccagctgttttccag	hUBC_4
hUBC	cagtgagtgtcttcacgaag	hUBC_5
hUBC	tcctggatctttgctttgac	hUBC_6
hUBC	cagggtagactctttctgga	hUBC_7
hUBC	cttcacgaagatctgcatcc	hUBC_8
hUBC	tcttggatctttgccttgac	hUBC_9
hUBC	cagtgagtgtcttcacgaag	hUBC_10
hUBC	tgacgttctcgatagtgtca	hUBC_11
hUBC	tccttgtcttggatctttgc	hUBC_12
hUBC	cagggtagactctttctgga	hUBC_13
hUBC	cttcacgaagatctgcatcc	hUBC_14
hUBC	agagtgatggtcttaccagt	hUBC_15
hUBC	tcttggatctttgccttgac	hUBC_16
hUBC	cttcacgaagatctgcatcc	hUBC_17
hUBC	agagtgatggtcttaccagt	hUBC_18

hUBC	tcttggatctttgccttgac	hUBC_19
hUBC	tgtttcccagcaaagatcaa	hUBC_20
hUBC	cttcacgaagatctgcatcc	hUBC_21
hUBC	agagtgatggtcttaccagt	hUBC_22
hUBC	tcttggatctttgccttgac	hUBC_23
hUBC	tgtttcccagcaaagatcaa	hUBC_24
hUBC	cttcacgaagatctgcatcc	hUBC_25
hUBC	agagtgatggtcttaccagt	hUBC_26
hUBC	tcttggatctttgccttgac	hUBC_27
hUBC	tgtttcccagcaaagatcaa	hUBC_28
hUBC	gacattctcgatggtgtcac	hUBC_29
hUBC	gggatgccttccttatcttg	hUBC_30
hUBC	tgtttcccagcaaagatcaa	hUBC_31
hUBC	agagtggactctttctggat	hUBC_32

Supplementary Table 4: Primers used to index ATAC sequencing libraries

primer name	sequence	index
N7001	CAAGCAGAAGACGGCATACGAGATTCGCCTTAGTCTCGTGGGCTCGGAGATGTG	TAAGGCGA
N7002	CAAGCAGAAGACGGCATACGAGATCTAGTACGGTCTCGTGGGCTCGGAGATGTG	CGTACTAG
N7003	CAAGCAGAAGACGGCATACGAGATTTCTGCCTGTCTCGTGGGCTCGGAGATGTG	AGGCAGAA
N7004	CAAGCAGAAGACGGCATACGAGATGCTCAGGAGTCTCGTGGGCTCGGAGATGTG	TCCTGAGC
N7005	CAAGCAGAAGACGGCATACGAGATAGGAGTCCGTCTCGTGGGCTCGGAGATGTG	GGACTCCT
N7006	CAAGCAGAAGACGGCATACGAGATCATGCCTAGTCTCGTGGGCTCGGAGATGTG	TAGGCATG
N7007	CAAGCAGAAGACGGCATACGAGATGTAGAGAGGTCTCGTGGGCTCGGAGATGTG	CTCTCTAC
N7008	CAAGCAGAAGACGGCATACGAGATCCTCTCTGGTCTCGTGGGCTCGGAGATGTG	CAGAGAGG
N7009	CAAGCAGAAGACGGCATACGAGATAGCGTAGCGTCTCGTGGGCTCGGAGATGTG	GCTACGCT
N7010	CAAGCAGAAGACGGCATACGAGATCAGCCTCGGTCTCGTGGGCTCGGAGATGTG	CGAGGCTG
N7011	CAAGCAGAAGACGGCATACGAGATTGCCTCTTGTCTCGTGGGCTCGGAGATGTG	AAGAGGCA
N7012	CAAGCAGAAGACGGCATACGAGATTCCTCTACGTCTCGTGGGCTCGGAGATGTG	GTAGAGGA
N5001	AATGATACGGCGACCACCGAGATCTACACTAGATCGCTCGTCGGCAGCGTCAGATGTGTAT	TAGATCGC
N5002	AATGATACGGCGACCACCGAGATCTACACCTCTCTATTCGTCGGCAGCGTCAGATGTGTAT	CTCTCTAT
N5003	AATGATACGGCGACCACCGAGATCTACACTATCCTCTTCGTCGGCAGCGTCAGATGTGTAT	TATCCTCT
N5004	AATGATACGGCGACCACCGAGATCTACACAGAGTAGATCGTCGGCAGCGTCAGATGTGTAT	AGAGTAGA
N5005	AATGATACGGCGACCACCGAGATCTACACGTAAGGAGTCGTCGGCAGCGTCAGATGTGTAT	GTAAGGAG
N5006	AATGATACGGCGACCACCGAGATCTACACACTGCATATCGTCGGCAGCGTCAGATGTGTAT	ACTGCATA
N5007	AATGATACGGCGACCACCGAGATCTACACAAGGAGTATCGTCGGCAGCGTCAGATGTGTAT	AAGGAGTA
N5008	AATGATACGGCGACCACCGAGATCTACACCTAAGCCTTCGTCGGCAGCGTCAGATGTGTAT	CTAAGCCT
N5009	AATGATACGGCGACCACCGAGATCTACACTGGAAATCTCGTCGGCAGCGTCAGATGTGTAT	TGGAAATC

N5010	AATGATACGGCGACCACCGAGATCTACACAACATGATTCGTCGGCAGCGTCAGATGTGTAT	AACATGAT
N5011	AATGATACGGCGACCACCGAGATCTACACTGATGAAATCGTCGGCAGCGTCAGATGTGTAT	TGATGAAA
N5012	AATGATACGGCGACCACCGAGATCTACACGTCGGACTTCGTCGGCAGCGTCAGATGTGTAT	GTCGGACT

Extended Statistical Information

Figure 2B

hi1: 5nM trametinib
lo1: 1nM trametinib
lohi1: 1nM trametinib → 5nM trametinib

```
Linear mixed-effects model fit by REML
  Data: data

Random effects:
 Formula: ~1 | exp
        (Intercept) Residual
StdDev:    6.220154 9.280895

Fixed effects:  colony ~ Name
 Correlation:
          (Intr) Name11
Namelo1   -0.587
Namelohi1 -0.587  0.500

Standardized Within-Group Residuals:
       Min         Q1        Med         Q3        Max
-1.9368868 -0.4828889  0.1401880  0.5105373  1.5202984

Number of Observations: 21
Number of Groups: 7

Shapiro-Wilk normality test

data:  residuals
W = 0.96928, p-value = 0.717

Levene's Test for Homogeneity of Variance (center = median)
      Df F value Pr(>F)
group  2  1.7003 0.2107
      18

$emmeans
 Name  emmean   SE df lower.CL upper.CL
 hi1     20.6 4.22  6     10.2     30.9
 lo1     67.6 4.22  6     57.2     77.9
 lohi1   59.3 4.22  6     49.0     69.6

Degrees-of-freedom method: containment
Confidence level used: 0.95

$contrasts
 contrast    estimate   SE df t.ratio p.value
 hi1 - lo1     -47.00 4.96 12  -9.474  <.0001
 hi1 - lohi1   -38.71 4.96 12  -7.804  <.0001
 lo1 - lohi1     8.29 4.96 12   1.670  0.1207

Degrees-of-freedom method: containment
P value adjustment: holm method for 3 tests
```

Figure 2E

a_bc30 - 5nM trametinib (replicate of c_bc30: cells from the same barcoding event but plated and treated separately from c_bc30 as a control of barcode overlap) - shown in Supplemental Figure 2D
b_bc30 - 1nM trametinib
c_bc30 - 5nM trametinib
d_bc30 - 1nM trametinib → 5nM trametinib

```
Linear mixed-effects model fit by REML
  Data: filtered_data
      AIC      BIC    logLik
  134.847 137.6718 -62.42351

Random effects:
 Formula: ~1 | Experiment
        (Intercept) Residual
StdDev:    7.600884 23.47272

Fixed effects:  Length ~ Name_stat
                  Value Std.Error DF  t-value p-value
(Intercept)      71.875  9.127675 10 7.874404   0e+00
Name_statb_bc30 104.125 14.374049 10 7.243958   0e+00
Name_statd_bc30  71.625 14.374049 10 4.982938   6e-04
 Correlation:
                (Intr) Nm_sttb_30
Name_statb_bc30 -0.525
Name_statd_bc30 -0.525  0.333

Standardized Within-Group Residuals:
       Min         Q1        Med         Q3        Max
-1.7606615 -0.4436539 -0.1288347  0.4518197  1.4284374

Number of Observations: 16
Number of Groups: 4

       Shapiro-Wilk normality test

data:  residuals(model, type = "pearson")
W = 0.9484, p-value = 0.4647

Levene's Test for Homogeneity of Variance (center = median)
      Df F value Pr(>F)
group  2  1.9419 0.1828
      13
$emmeans
 Name_stat emmean    SE df lower.CL upper.CL
 ac_bc30     71.9  9.13  3     42.8      101
 b_bc30     176.0 12.30  3    136.7      215
 d_bc30     143.5 12.30  3    104.2      183

Degrees-of-freedom method: containment
Confidence level used: 0.95

$contrasts
 contrast         estimate   SE df t.ratio p.value
 ac_bc30 - b_bc30   -104.1 14.4 10  -7.244  0.0001
 ac_bc30 - d_bc30    -71.6 14.4 10  -4.983  0.0011
 b_bc30 - d_bc30      32.5 16.6 10   1.958  0.0787

Degrees-of-freedom method: containment
P value adjustment: holm method for 3 tests
```

Figure 2G

hi1: 5nM trametinib
hi2: 5nM trametinib + JNK-IN-8
lo1: 1nM trametinib → 1nM trametinib
lo2: 1nM trametinib + 1μM JNK-IN-8 → 1nM trametinib
lohi1: 1nM trametinib → 5nM trametinib
lohi2: 1nM trametinib + 1μM JNK-IN-8 → 5nM trametinib

```
Linear mixed-effects model fit by REML
  Data: data

Random effects:
 Formula: ~1 | exp
        (Intercept) Residual
StdDev:    7.152311 6.566751

Fixed effects:  colony ~ Name
 Correlation:
          (Intr) Nameh2 Namel1 Namel2 Namlh1
Namehi2   -0.478
Namelo1   -0.478  0.500
Namelo2   -0.478  0.500  0.500
Namelohi1 -0.478  0.500  0.500  0.500
Namelohi2 -0.478  0.500  0.500  0.500  0.500

Standardized Within-Group Residuals:
        Min          Q1         Med          Q3         Max
-1.65806047 -0.56812988 -0.03390281  0.42846083  1.22959963

Shapiro-Wilk normality test

data:  residuals
W = 0.94632, p-value = 0.5839

Levene's Test for Homogeneity of Variance (center = median)
      Df F value Pr(>F)
group  5   0.555 0.7324
      12


Degrees-of-freedom method: containment
Confidence level used: 0.95

$contrasts
 contrast      estimate   SE df t.ratio p.value
 hi1 - hi2        11.00 5.36 10   2.052  0.4040
 hi1 - lo1       -33.33 5.36 10  -6.217  0.0011
 hi1 - lo2       -30.33 5.36 10  -5.657  0.0017
 hi1 - lohi1     -29.00 5.36 10  -5.409  0.0021
 hi1 - lohi2       2.00 5.36 10   0.373  1.0000
 hi2 - lo1       -44.33 5.36 10  -8.268  0.0001
 hi2 - lo2       -41.33 5.36 10  -7.709  0.0002
 hi2 - lohi1     -40.00 5.36 10  -7.460  0.0003
 hi2 - lohi2      -9.00 5.36 10  -1.679  0.6208
 lo1 - lo2         3.00 5.36 10   0.560  1.0000
 lo1 - lohi1       4.33 5.36 10   0.808  1.0000
 lo1 - lohi2      35.33 5.36 10   6.590  0.0007
 lo2 - lohi1       1.33 5.36 10   0.249  1.0000
 lo2 - lohi2      32.33 5.36 10   6.030  0.0013
 lohi1 - lohi2    31.00 5.36 10   5.782  0.0016

Degrees-of-freedom method: containment
P value adjustment: holm method for 15 tests
```

Figure 3B

CY3: LTBP1
A594: FKBP5
CY6: TGM2

Sample_identifier1: DMSO (d0-d3)

Sample_identifier2: dex (d0-d3)
Sample_identifier3: dex (d0-d3) → DMSO (d3-d17)
Sample_identifier4: DMSO (d0-d3) → tram (d3-d17)
Sample_identifier5: dex (d0-d3) → tram (d3-d17)
Sample_identifier6: dex (d0-d2) → dex + JNK-IN-8 (d2-d3) → tram (d3-d17)

```
Shapiro-Wilk test for raw CY3 data: p = 4.7637e-08
Shapiro-Wilk test for raw A594 data: p = 0.00020842
Shapiro-Wilk test for raw CY5 data: p = 2.529e-06
```

```
=== COMPREHENSIVE RESULTS FOR CY3 ===
Test statistics (z-ratios): -1.265, 0.717, -0.519, -4.597, -1.685, 1.982, 0.746, -3.331, -0.419, -1.236, -5.314,
-2.401, -4.078, -1.166, 2.912
Degrees of freedom: Inf, Inf, Inf, Inf, Inf, Inf, Inf, Inf, Inf, Inf, Inf, Inf, Inf, Inf, Inf
Exact p-values (Holm-adjusted): 1.0000000, 1.0000000, 1.0000000, 6.009e-05, 0.7365672, 0.4272185, 1.0000000,
0.0103664, 1.0000000, 1.0000000, 1.612e-06, 0.1633167, 0.0005913, 1.0000000, 0.0394843
95% Confidence intervals (Holm-adjusted):
 contrast                                 estimate      SE  df asymp.LCL asymp.UCL
 sample_identifier1 - sample_identifier2 -0.00619 0.00489 Inf -0.020538   0.00817
 sample_identifier1 - sample_identifier3  0.00350 0.00489 Inf -0.010847   0.01786
 sample_identifier1 - sample_identifier4 -0.00254 0.00489 Inf -0.016889   0.01181
 sample_identifier1 - sample_identifier5 -0.02248 0.00489 Inf -0.036827  -0.00812
 sample_identifier1 - sample_identifier6 -0.00824 0.00489 Inf -0.022588   0.00611
 sample_identifier2 - sample_identifier3  0.00969 0.00489 Inf -0.004660   0.02404
 sample_identifier2 - sample_identifier4  0.00365 0.00489 Inf -0.010703   0.01800
 sample_identifier2 - sample_identifier5 -0.01629 0.00489 Inf -0.030641  -0.00194
 sample_identifier2 - sample_identifier6 -0.00205 0.00489 Inf -0.016402   0.01230
 sample_identifier3 - sample_identifier4 -0.00604 0.00489 Inf -0.020394   0.00831
 sample_identifier3 - sample_identifier5 -0.02598 0.00489 Inf -0.040332  -0.01163
 sample_identifier3 - sample_identifier6 -0.01174 0.00489 Inf -0.026093   0.00261
 sample_identifier4 - sample_identifier5 -0.01994 0.00489 Inf -0.034289  -0.00559
 sample_identifier4 - sample_identifier6 -0.00570 0.00489 Inf -0.020051   0.00865
 sample_identifier5 - sample_identifier6  0.01424 0.00489 Inf -0.000113   0.02859
Confidence level used: 0.95
Conf-level adjustment: bonferroni method for 15 estimates
Effect sizes (z-ratios): -1.265, 0.717, -0.519, -4.597, -1.685, 1.982, 0.746, -3.331, -0.419, -1.236, -5.314,
-2.401, -4.078, -1.166, 2.912
Significant comparisons (p < 0.05):
  sample_identifier1 - sample_identifier5: z = -4.597, p = 6.009e-05
  sample_identifier2 - sample_identifier5: z = -3.331, p = 0.01037
  sample_identifier3 - sample_identifier5: z = -5.314, p = 1.612e-06
  sample_identifier4 - sample_identifier5: z = -4.078, p = 0.0005913
  sample_identifier5 - sample_identifier6: z = 2.912, p = 0.03948
Summary for CY3 :
  Total comparisons: 15
  Significant comparisons: 5
  Range of estimates: -0.025981 to 0.014239
=== COMPREHENSIVE RESULTS FOR A594 ===
Test statistics (z-ratios): -3.594, 0.124, 0.033, -5.543, -0.114, 3.718, 3.627, -1.949, 3.48, -0.091, -5.667,
-0.238, -5.577, -0.148, 5.429
Degrees of freedom: Inf, Inf, Inf, Inf, Inf, Inf, Inf, Inf, Inf, Inf, Inf, Inf, Inf, Inf, Inf
Exact p-values (Holm-adjusted): 0.002934, 1.000000, 1.000000, 3.862e-07, 1.000000, 0.002210, 0.002865, 0.358697,
0.004017, 1.000000, 2.177e-07, 1.000000, 3.433e-07, 1.000000, 6.799e-07
95% Confidence intervals (Holm-adjusted):
 contrast                                 estimate      SE  df asymp.LCL asymp.UCL
 sample_identifier1 - sample_identifier2 -0.017085 0.00475 Inf  -0.03104  -0.00313
 sample_identifier1 - sample_identifier3  0.000590 0.00475 Inf  -0.01336   0.01454
 sample_identifier1 - sample_identifier4  0.000159 0.00475 Inf  -0.01380   0.01411
 sample_identifier1 - sample_identifier5 -0.026353 0.00475 Inf  -0.04031  -0.01240
 sample_identifier1 - sample_identifier6 -0.000542 0.00475 Inf  -0.01450   0.01341
 sample_identifier2 - sample_identifier3  0.017675 0.00475 Inf   0.00372   0.03163
 sample_identifier2 - sample_identifier4  0.017244 0.00475 Inf   0.00329   0.03120
 sample_identifier2 - sample_identifier5 -0.009268 0.00475 Inf  -0.02322   0.00469
 sample_identifier2 - sample_identifier6  0.016543 0.00475 Inf   0.00259   0.03050
 sample_identifier3 - sample_identifier4 -0.000431 0.00475 Inf  -0.01439   0.01352
 sample_identifier3 - sample_identifier5 -0.026943 0.00475 Inf  -0.04090  -0.01299
 sample_identifier3 - sample_identifier6 -0.001133 0.00475 Inf  -0.01509   0.01282
 sample_identifier4 - sample_identifier5 -0.026513 0.00475 Inf  -0.04047  -0.01256
```

```
 sample_identifier4 - sample_identifier6 -0.000702 0.00475 Inf  -0.01466   0.01325
 sample_identifier5 - sample_identifier6  0.025811 0.00475 Inf   0.01186   0.03977
Confidence level used: 0.95
Conf-level adjustment: bonferroni method for 15 estimates
Effect sizes (z-ratios): -3.594, 0.124, 0.033, -5.543, -0.114, 3.718, 3.627, -1.949, 3.48, -0.091, -5.667,
-0.238, -5.577, -0.148, 5.429
Significant comparisons (p < 0.05):
  sample_identifier1 - sample_identifier2: z = -3.594, p = 0.002934
  sample_identifier1 - sample_identifier5: z = -5.543, p = 3.862e-07
  sample_identifier2 - sample_identifier3: z = 3.718, p = 0.00221
  sample_identifier2 - sample_identifier4: z = 3.627, p = 0.002865
  sample_identifier2 - sample_identifier6: z = 3.480, p = 0.004017
  sample_identifier3 - sample_identifier5: z = -5.667, p = 2.177e-07
  sample_identifier4 - sample_identifier5: z = -5.577, p = 3.433e-07
  sample_identifier5 - sample_identifier6: z = 5.429, p = 6.799e-07
Summary for A594 :
  Total comparisons: 15
  Significant comparisons: 8
  Range of estimates: -0.026943 to 0.025811
=== COMPREHENSIVE RESULTS FOR CY5 ===
Test statistics (z-ratios): -1.165, 0.039, -0.941, -5.567, -1.996, 1.204, 0.225, -4.402, -0.831, -0.98, -5.606,
-2.035, -4.626, -1.055, 3.571
Degrees of freedom: Inf, Inf, Inf, Inf, Inf, Inf, Inf, Inf, Inf, Inf, Inf, Inf, Inf, Inf, Inf
Exact p-values (Holm-adjusted): 1.0000000, 1.0000000, 1.0000000, 3.623e-07, 0.4183128, 1.0000000, 1.0000000,
0.0001288, 1.0000000, 1.0000000, 3.102e-07, 0.4183128, 4.837e-05, 1.0000000, 0.0039118
95% Confidence intervals (Holm-adjusted):
 contrast                                estimate      SE  df asymp.LCL asymp.UCL
 sample_identifier1 - sample_identifier2 -0.009114 0.00782 Inf  -0.03207   0.01384
 sample_identifier1 - sample_identifier3  0.000304 0.00782 Inf  -0.02265   0.02326
 sample_identifier1 - sample_identifier4 -0.007359 0.00782 Inf  -0.03031   0.01560
 sample_identifier1 - sample_identifier5 -0.043541 0.00782 Inf  -0.06650  -0.02059
 sample_identifier1 - sample_identifier6 -0.015613 0.00782 Inf  -0.03857   0.00734
 sample_identifier2 - sample_identifier3  0.009419 0.00782 Inf  -0.01354   0.03237
 sample_identifier2 - sample_identifier4  0.001756 0.00782 Inf  -0.02120   0.02471
 sample_identifier2 - sample_identifier5 -0.034427 0.00782 Inf  -0.05738  -0.01147
 sample_identifier2 - sample_identifier6 -0.006498 0.00782 Inf  -0.02945   0.01646
 sample_identifier3 - sample_identifier4 -0.007663 0.00782 Inf  -0.03062   0.01529
 sample_identifier3 - sample_identifier5 -0.043846 0.00782 Inf  -0.06680  -0.02089
 sample_identifier3 - sample_identifier6 -0.015917 0.00782 Inf  -0.03887   0.00704
 sample_identifier4 - sample_identifier5 -0.036183 0.00782 Inf  -0.05914  -0.01323
 sample_identifier4 - sample_identifier6 -0.008254 0.00782 Inf  -0.03121   0.01470
 sample_identifier5 - sample_identifier6  0.027929 0.00782 Inf   0.00497   0.05088
Confidence level used: 0.95
Conf-level adjustment: bonferroni method for 15 estimates
Effect sizes (z-ratios): -1.165, 0.039, -0.941, -5.567, -1.996, 1.204, 0.225, -4.402, -0.831, -0.98, -5.606,
-2.035, -4.626, -1.055, 3.571
Significant comparisons (p < 0.05):
  sample_identifier1 - sample_identifier5: z = -5.567, p = 3.623e-07
  sample_identifier2 - sample_identifier5: z = -4.402, p = 0.0001288
  sample_identifier3 - sample_identifier5: z = -5.606, p = 3.102e-07
  sample_identifier4 - sample_identifier5: z = -4.626, p = 4.837e-05
  sample_identifier5 - sample_identifier6: z = 3.571, p = 0.003912
Summary for CY5 :
  Total comparisons: 15
  Significant comparisons: 5
  Range of estimates: -0.043846 to 0.027929
```

Supplemental Figure 2A

plxhi - 1µM vemurafenib
plxlo - 100nM vemurafenib
plxlohi - 100nM vemurafenib → 1µM vemurafenib

```
Linear mixed-effects model fit by REML
  Data: data

Random effects:
```

```
 Formula: ~1 | exp
        (Intercept) Residual
StdDev:    5.730038 5.317685

Fixed effects:  colony ~ Name 
 Correlation: 
            (Intr) Namplxl
Nameplxlo   -0.481        
Nameplxlohi -0.481  0.500 

Standardized Within-Group Residuals:
         Min           Q1          Med           Q3          Max 
-1.110757861 -0.392337573 -0.001318499  0.234501566  1.503095434 

Number of Observations: 9
Number of Groups: 3 

       Shapiro-Wilk normality test

data:  residuals
W = 0.96139, p-value = 0.8127

Levene's Test for Homogeneity of Variance (center = median)
      Df F value Pr(>F)
group  2  1.3795 0.3214
       6               
$emmeans
 Name    emmean   SE df lower.CL upper.CL
 plxhi     9.67 4.51  2    -9.75     29.1
 plxlo    64.00 4.51  2    44.58     83.4
 plxlohi  59.00 4.51  2    39.58     78.4

Degrees-of-freedom method: containment 
Confidence level used: 0.95 

$contrasts
 contrast        estimate   SE df t.ratio p.value
 plxhi - plxlo      -54.3 4.34  4 -12.514  0.0007
 plxhi - plxlohi    -49.3 4.34  4 -11.362  0.0007
 plxlo - plxlohi      5.0 4.34  4   1.152  0.3136

Degrees-of-freedom method: containment 
P value adjustment: holm method for 3 tests 
```
