# Supplementary material for: AP-1 mediates cellular adaptation and memory formation
Source: Nat Commun. 2026 Mar 20;17:4265. doi: 10.1038/s41467-026-70862-w (PMC13168276; doi:10.1038/s41467-026-70862-w)
Supplement: Supplementary file 2 — Reporting Summary [file 41467_2026_70862_MOESM2_ESM.pdf]

nature portfolio

Corresponding author(s): Arjun Raj

Last updated by author(s): Dec 1, 2025

Reporting Summary

Nature Portfolio wishes to improve the reproducibility of the work that we publish. This form provides structure for consistency and transparency in reporting. For further information on Nature Portfolio policies, see our Editorial Policies and the Editorial Policy Checklist.

Statistics

For all statistical analyses, confirm that the following items are present in the figure legend, table legend, main text, or Methods section.

n/a	Confirmed	
☐	☒	The exact sample size (*n*) for each experimental group/condition, given as a discrete number and unit of measurement
☐	☒	A statement on whether measurements were taken from distinct samples or whether the same sample was measured repeatedly
☐	☒	The statistical test(s) used AND whether they are one- or two-sided<br>*Only common tests should be described solely by name; describe more complex techniques in the Methods section.*
☐	☒	A description of all covariates tested
☐	☒	A description of any assumptions or corrections, such as tests of normality and adjustment for multiple comparisons
☐	☒	A full description of the statistical parameters including central tendency (e.g. means) or other basic estimates (e.g. regression coefficient) AND variation (e.g. standard deviation) or associated estimates of uncertainty (e.g. confidence intervals)
☐	☒	For null hypothesis testing, the test statistic (e.g. *F*, *t*, *r*) with confidence intervals, effect sizes, degrees of freedom and *P* value noted<br>*Give P values as exact values whenever suitable.*
☒	☐	For Bayesian analysis, information on the choice of priors and Markov chain Monte Carlo settings
☒	☐	For hierarchical and complex designs, identification of the appropriate level for tests and full reporting of outcomes
☒	☐	Estimates of effect sizes (e.g. Cohen's *d*, Pearson's *r*), indicating how they were calculated

Our web collection on statistics for biologists contains articles on many of the points above.

Software and code

Policy information about availability of computer code

Data collection	No commercial, open source or custom code was used to collect data in this study.

Data analysis

All code generated and used for data analysis in this paper is available at https://github.com/arjunrajlaboratory/cellularmemory. Image analysis was conducted using NimbusImage (https://github.com/Kitware/UPennContrast) unless otherwise noted. Cell segmentation was performed manually due to irregular cell shapes and overlapping in resistant colonies. For colony segmentation, we used SegmentAnything (https://github.com/facebookresearch/segment-anything), a built-in function of NimbusImage. Colonies were tracked across time points throughout the experiment to distinguish between single-cell-derived colonies and merged colonies. For time-lapse analysis of naive cells, SegmentAnything was used to annotate individual cells, tracking them every 8 hours. Fluorescence intensity calculations varied based on imaging conditions. For 10x magnification images (Nikon scope), colony fluorescence was calculated as the 90th percentile of the colony's fluorescence minus the median fluorescence of randomly selected colony-free background areas (Figure 4C). For 4x magnification images (Incucyte S3), individual cell fluorescence was calculated as the mean fluorescence of the cell annotation minus the median fluorescence of a 10-pixel annulus around the annotation, while whole colony fluorescence was calculated as the mean of all pixels above the median fluorescence within the colony annotation, minus the annotation's median fluorescence in order to account of spaces between cells within a colony (Figure 4E). Bulk fluorescence intensity analysis for AP-1 reporter controls imaged on Incucyte S3 was conducted directly on the Incucyte S3 software by calculating the fluorescence intensity (GCU or RCU per well, for green and red signals, respectively), normalized to the % confluency of the well. For RNA smFISH experiments, RNA spots were identified using Piscis, a deep-learning algorithm (https://github.com/zjniu/Piscis)67 (model 20230905). Spot counts were normalized to the cell area and displayed as spots per micron². In densely packed cell cases, Cellpose2 was used to segment DAPI-stained nuclei and Piscis-detected spots were associated with the nearest nucleus, displayed as spots per nucleus. Image annotation data were exported as CSVs for further processing and graphing in R. NimbusImage annotations and connections were also exported in JSON format.

For manuscripts utilizing custom algorithms or software that are central to the research but not yet described in published literature, software must be made available to editors and reviewers. We strongly encourage code deposition in a community repository (e.g. GitHub). See the Nature Portfolio guidelines for submitting code & software for further information.

Data

Policy information about availability of data

All manuscripts must include a data availability statement. This statement should provide the following information, where applicable:

- Accession codes, unique identifiers, or web links for publicly available datasets
- A description of any restrictions on data availability
- For clinical datasets or third party data, please ensure that the statement adheres to our policy

Source data are provided with this paper. The next-generation sequencing data generated in this study have been deposited in the GEO database under accession codes GSE311825, GSE311827, GSE311829. Re-analysis of published datasets available at GSE97682 & GSE161300) All raw and processed data are accessible here (https://www.dropbox.com/scl/fo/mx4ef9lvs3bzw8ms18jwt/ABv0e0o_njpT0ooPzNNbgC8?rlkey=0vhml03taajkdkoma0azg4k5t&dl=0).

Research involving human participants, their data, or biological material

Policy information about studies with human participants or human data. See also policy information about sex, gender (identity/presentation), and sexual orientation and race, ethnicity and racism.

Reporting on sex and gender	N/A
Reporting on race, ethnicity, or other socially relevant groupings	N/A
Population characteristics	N/A
Recruitment	N/A
Ethics oversight	N/A

Note that full information on the approval of the study protocol must also be provided in the manuscript.

Field-specific reporting

Please select the one below that is the best fit for your research. If you are not sure, read the appropriate sections before making your selection.

☒ Life sciences ☐ Behavioural & social sciences ☐ Ecological, evolutionary & environmental sciences

For a reference copy of the document with all sections, see nature.com/documents/nr-reporting-summary-flat.pdf

Life sciences study design

All studies must disclose on these points even when the disclosure is negative.

Sample size	Sample sizes were determined based on field standards, with n≥2 for most experimental conditions. The majority of experiments were conducted with n≥3 biological replicates. In specific instances [list specific experiments/conditions], n=1 was used due to resource constraints.
Data exclusions	Data were excluded from analysis when experiments failed quality control criteria, including contamination or technical failures identified

Data exclusions	through independent verification. Additional experiments beyond the scope of the current study were excluded from this analysis. Individual replicates are displayed in figures when possible to provide transparency regarding biological variability. Even when replicates are not shown for data visualization simplicity, all raw data, processed data, and analysis code are available in the accompanying data repository.
Replication	In order to ensure the reproducibility of the findings, several key experiments were repeated by 2-3 different lab members across various times with various cell lines and clones.
Randomization	In each experiment, each biological replicate was initiated with cells from the same passage and culture to ensure uniform starting populations across all experimental conditions. For RNA-seq and ATAC-seq experiments, sample processing order was determined by random selection to control for potential batch effects and processing bias.
Blinding	Blinding was not feasible for cell culture experiments due to the requirement for manual intervention during drug treatments and media changes. However, for RNA-seq and ATAC-seq experiments, sample processing order was randomized to control for potential batch effects and processing bias.

Reporting for specific materials, systems and methods

We require information from authors about some types of materials, experimental systems and methods used in many studies. Here, indicate whether each material, system or method listed is relevant to your study. If you are not sure if a list item applies to your research, read the appropriate section before selecting a response.

Materials & experimental systems

n/a	Involved in the study
☒	☐ Antibodies
☐	☒ Eukaryotic cell lines
☒	☐ Palaeontology and archaeology
☒	☐ Animals and other organisms
☒	☐ Clinical data
☒	☐ Dual use research of concern
☒	☐ Plants

Methods

n/a	Involved in the study
☒	☐ ChIP-seq
☐	☒ Flow cytometry
☒	☐ MRI-based neuroimaging

Eukaryotic cell lines

Policy information about cell lines and Sex and Gender in Research

Cell line source(s)	WM989 cell lines and subsequent clones are from the laboratory of Meenhard Herlyn. H358 cells were purchased directly from ATCC.
Authentication	WM989 melanoma cells from the laboratory of Meenhard Herlyn were validated by DNA short tandem repeat (STR) microsatellite fingerprinting at the Wistar Institute. H358 cells were purchased directly from ATCC but not re-authenticated once received.
Mycoplasma contamination	all cell lines used have tested negative for mycoplasma contamination
Commonly misidentified lines (See ICLAC register)	We are not using a cell line that is on this list.

Plants

Seed stocks	N/A
Novel plant genotypes	N/A
Authentication	N/A

Flow Cytometry

Plots

Confirm that:

☒ The axis labels state the marker and fluorochrome used (e.g. CD4-FITC).

☒ The axis scales are clearly visible. Include numbers along axes only for bottom left plot of group (a 'group' is an analysis of identical markers).

☒ All plots are contour plots with outliers or pseudocolor plots.

☒ A numerical value for number of cells or percentage (with statistics) is provided.

Methodology

Sample preparation	We used 0.05% trypsin-EDTA to detach barcoded cells from the plate and subsequently neutralize the trypsin with the corresponding medium. We then pelleted the cells, performed a wash with 1× DPBS, and resuspended them again in 1× DPBS. Cells were sorted on a BD FACSJazz machine (BD Biosciences) with a 100 µm nozzle, gated for positive GFP signal and singlets. Sorted cells were then centrifuged to remove the supernatant medium containing PBS, and replated with the appropriate cell culture medium.
Instrument	Cells were sorted on a BD FACSJazz machine (BD Biosciences)
Software	The default software on the BD FACSJazz was used
Cell population abundance	the population of interest (GFP+ cells) were approximately 20% of the population. These cells were later cultured and the viral construct containing the GFP and a DNA barcode were sequenced
Gating strategy	FSC/SSC were used to identify a live cell population, then FSC/trigger pulse width were used to identify singlets, an untransduced control was used to set the gate for the GFP+ cells

☒ Tick this box to confirm that a figure exemplifying the gating strategy is provided in the Supplementary Information.
