# Supplementary material for: AP-1 mediates cellular adaptation and memory formation
Source: Nat Commun. 2026 Mar 20;17:4265. doi: 10.1038/s41467-026-70862-w (PMC13168276; doi:10.1038/s41467-026-70862-w)
Supplement: Supplementary file 3 [file 41467_2026_70862_MOESM3_ESM.pdf]

nature portfolio

Peer Review File

AP-1 Mediates Cellular Adaptation and Memory Formation

Corresponding Author: Professor Arjun Raj

This file contains all reviewer reports in order by version, followed by all author rebuttals in order by version.

Version 0:

Reviewer comments:

Reviewer #1

(Remarks to the Author)
This manuscript by Dr. Raj and colleagues is a complex exploration of cellular memory in the context of therapy resistance in cancer cells. The overarching aim is to determine whether resistance to treatments arises from selection and further adaptation to treatment stress, or, in contrast, if resistance is predefined in specific cells within the tumor population. The authors used a single melanoma cell line treated primarily with the BRAFV600E inhibitor vemurafenib as a model system. They provide evidence supporting an adaptive cellular mechanism rather than a pre-existing resistant cell population prior to treatment initiation. Gene expression and ATAC-seq data suggest a primed state of resistance, with minor changes in expression and chromatin accessibility that become more pronounced in resistant cells. Additionally, AP-1 transcription factor binding motifs are enriched in the open chromatin regions in resistant cells. Further experiments suggest that cellular adaptation to the treatment occurs in a dose-dependent manner.

In the second part of the manuscript, the authors investigate whether specific gene expression changes during the adaptation of resistant cells are the result of a predefined gene regulatory program or an acquired adaptation, referred to as "memory," the latter being the proposed mechanism. Finally, they generated cell lines expressing a reporter system with canonical AP-1 binding sites upstream of a minimal promoter that drives expression of either EGFP or mCherry. Using this system, they propose that "the epigenetic memory formed by the addition of trametinib is encoded in cis."

I found this manuscript challenging to read and follow. The text is concise, but the language is occasionally too grandiose, and some conclusions seem overstated. Figure legends are also too brief, often missing important details. While certain aspects of this study are convincing (I particularly appreciate the results in Figure 4d and the experimental approach in Figure 2d), the study’s main conclusions are based on experiments conducted in a single cell line and with only one type of treatment. Additionally, the authors do not convincingly demonstrate epigenetic memory as claimed; instead, they merely show that AP-1 sites are accessible, which alone is insufficient to assert that epigenetic memory governs resistance.

Honestly, I struggled to fully understand Figure 3, despite reviewing it multiple times over different days. The text does not aid comprehension, and some gene expression differences, such as those of LTBP1 and TGM2, are unclear (e.g., I don't observe their upregulation upon trametinib treatment). Moreover, there is notable variability in FKBP5, LTBP1, and TGM2 between replicates. Along these lines, some conclusions are based on changes in the expression of a single gene (FKBP5) in one cell line and under one treatment condition.

Overall, I find this study to be too preliminary, with conclusions that are largely overstated.

(Remarks on code availability)

Reviewer #2

(Remarks to the Author)
The manuscript presents an elegant and thoughtfully designed study on cellular adaptation, a novel and intriguing perspective in the field of cancer therapy resistance. This work contributes significantly to the shifting away from the traditional binary model of resistance, often attributed to selection of pre-existing or acquired resistant cells. The authors elegantly demonstrate the adaptive process where cells respond dynamically to therapy by incorporating previous

experiences (drug exposure) into epigenetic memories.

The use of dual-color reporter experiments is particularly creative and compelling, reinforcing the concept of adaptive cellular behavior in a nuanced manner. The finding that the transcription factor AP-1 plays a central role in regulating these adaptive behaviors and establishing a "memory" under therapeutic stress adds an intriguing layer of mechanistic understanding, and aligns well with previous work in cancer and other fields.

Technically, the manuscript is robust, and the experiments are logically arranged, well executed, and thoughtful in design. This paper holds significance for the field and is worthy of publication. Below are specific issues that should be addressed in terms of contextualization and clarity.

Regarding potential mechanisms for chromatin modifications: a recent study has shown at single-cell resolution and lineage barcoding that cells initially surviving therapy ('persisters') are primed with H3K4me3 and H3K27me3, thus enhancing the potential for resistance (Marsolier et al. Nat Genetics, 2022: PMID: PMC7612638). This could be mentioned in the discussion as possible cis- epigenetic signals. Also, our own study, (França et al., Nature, 2024: PMID: 38987605) used a dose escalation approach, as employed here, and showed that previous exposure to lower doses enabled the emergence of resistant populations to higher doses in an ovarian cancer model — a phenotypic observation that parallels this work. This could be acknowledged and discussed in support of the authors' findings. In addition, transcriptional and chromatin changes that do not resemble initial states ('persister' cells) with increased AP-1 accessibility were associated with higher resistance in this work, suggesting recall of an epigenetic memory after drug holiday and re-exposure. In the present study, the authors have uniquely demonstrated some of these processes with greater resolution and detail using single-clone tracking and cellular barcoding, with very interesting results. Addressing these recent findings would provide support and contextualization.

In Figure 1, further clarification is needed regarding the transcriptomic data. Specifically, while the authors cite their 2021 paper, additional details on how the transcriptomic and chromatin profiles of the 'non-primed', 'primed' and 'resistant' cells were defined a priori. A better clarification on how these populations were defined is required.

'Full resistance' could be better defined in this context. Do the authors consider 'resistant' as cells that are able to survive drug exposure, to proliferate under treatment, or through a quantitative measure such as the half-maximal inhibitory drug concentration (IC50)? A more explicit and, if possible, quantitative definition of resistance in this model would provide additional clarity.

In several figures showing ATAC-seq profiles (ex: Fig. 1e) the colorbar is not labeled.

Gustavo Starvaggi França and Itai Yanai

(Remarks on code availability)

Reviewer #3

(Remarks to the Author)
The paper by Li, et al continues the Raj group's in-depth investigation of the cellular resistance and memory effect in models of cancer. Here the focus is on buildup of cellular memory and the involvement of AP-1 in mediating it. The authors' model suggests that following stress (in this paper it is drug exposure), gene expression that may have been linked with exposure, but also transient or incidental, can become fixated through a mechanism that is dependent on AP-1 activity. Such expression can be stably propagated in affected clones. It may can lead to adaptation, for example in scenarios of gradual dose increase. And it is shown to be facilitated in-cis, according to experiments separating clonal memory effects in two independent loci.
This is a really interesting, original and important work. The experimental design, data collected and analysis are all of top quality. I strongly support its timely publication.
I'm listing here some points for potential consideration by the authors. These can be used to improve presentation/clarity and add some potential insights, but I do not believe the paper should be expanded significantly or that new data should be added. The questions addressed here are going to be followed up by the authors and by others, and the present study represent an important advance. I provide some suggestions for adding evidence on the mechanisms for in-cis memory using more ATAC analysis, but in my opinion, if the authors decide they wish to keep the arguments here at a higher level, then this can be accepted.
Points for consideration:
1) Primed, resistant and adapted cells. The paper is based on a rather sophisticated, and potentially difficult to understand distinction cell priming and adaptation. These are complementary ideas which may be perceived as contradicting (e.g. one student asked: if only primed cells can adapt, is this really adaptation..?). The authors schematics in Figure 1 is perhaps not highlighting the most important aspects of this distinction, as it seems that the "adaptation" phase is just a direct consequence of the original response. It should be better clarified – a) that the altered state can persist after removing the stimulation. b) that it is possible that not all "primed" cells can adapt. If the author can further come up with a more uniform schematics for the experiments in Figure 2-3, it would be very helpful.
2) Describing complex experiments. I believe more subheadings/sections in the results, breaking the experimental work into smaller chunks with clear conclusions for each step, can be very helpful. The current experimental narrative is beautiful, but

require quite some effort for following the complex logic in relatively long series of arguments – it is unclear if typical readers will naturally go through this.
I did like the usage of additional text to explain supplementary figures (beyond legends). I hope this can be accommodated by the journal guidelines – it is the right thing to do!
3) In cis-memory and ATAC. ATAC analysis can probably provide significantly more insight. ChromVAR motif plots in Figure 1 and Figure S4 only show broad statistical behaviors that can be linked with (families of) TFs. Identifying accessibility peaks and clustering these to identify the main modes in the data can provide a much more direct read out. I realize this may be a domain the authors wish to follow up thoroughly later, which I can understand and support, but showing the ATAC data without even minimal in-cis analysis seems at odds with the very systematic approach to experiments that is represented in all other parts of this study. Possible points for consideration (3.1 seems like a must, the others optional at this stage):
3.1 Quantitative vs. hierarchical epigenetic programming – are there specific clusters of loci (not motifs!) that are becoming accessible only following adaptation, and clusters that become accessible already in the primed state, such that the average generate the gradual motif footprints shown now? Or is it gradual at the level of individual loci?
3.2 TSS, proximal, distal behaviors – would adaptation occur mostly at TSSs (probably not)? If it is also detected away from TSSs, are there specific chromosomal domains showing adaptation? For loci with potential AP-1 in-cis specificity, would the chromosomal context be distributed differently?
3.3 linkage to gene expression and feedback – How many of the adapted hotspots can be reasonably linked with a transcriptionally regulated TSS? (e.g. are within the same TAD or within e.g. 100kb)? Can the authors identify a group of ATAC peaks that are particularly isolated from any discernible transcriptional regulation? This will be really important and interesting in the context of feedback memory vs. in-cis transmission – concepts that are discussed a bit in the paper, but without much evidence.
3.4 Activated and repressive chromatin. One should identify TSSs or regulatory elements that are losing accessibility during adaptation/selection. If there are very few, it should be quantified. If not, this should be further characterized and discussed. Points for consideration: linkage with transcription, gradual decay, and the transmission/propagation of these changes in resistant clone (is repression only transient, and cannot be memorized? Is it linked with AP-1? An indirect consequence of Ap-1 mediated activation? If the argument is that there are _no_ accessibility hotspots that are being decreasing in intensity following adaptation, it will be quite surprising and need some better statistical quantification.
Minor atac points:
- The color scale of chromvar should be labled.
- Enrichment of CTCF motifs is hard to explain
- IT will be helpful to keep the rows (motifs) in the analysis using the same order across Fig 1 and S4 panels – for comparison.
4) proliferation and memory decay. It would be important to understand better memory decay and the directionality of the response to stress as analyzed here. If clones do maintain an adapted state for a month as briefly mentioned here, is it suggested “switching back” to the ground state is sufficiently rare to be ignored? What is the degree of stochasticity within the adapted clonal state, and can the memory be eliminated by another stress? These are points for discussion, mostly important in order to understand whether homeostasis or directional adaptation is the main mode to be expected within a tumor cell population,

(Remarks on code availability)

Reviewer #4

(Remarks to the Author)
I co-reviewed this manuscript with one of the reviewers who provided the listed reports. This is part of the Nature Communications initiative to facilitate training in peer review and to provide appropriate recognition for Early Career Researchers who co-review manuscripts.

(Remarks on code availability)

Version 1:

Reviewer comments:

Reviewer #1

(Remarks to the Author)
The authors did a great job addressing my main concerns. I do not have further comments.

(Remarks on code availability)
-

Reviewer #2

(Remarks to the Author)
The authors have done an admirable and professional job in revising the manuscript on all of the points raised across the reviews. We believe it is now ready to be accepted for publication.

(Remarks on code availability)

Reviewer #3

(Remarks to the Author)
The answers to our concerns make sense.

No additional comments

(Remarks on code availability)
No comments

Reviewer #4

(Remarks to the Author)
I co-reviewed this manuscript with one of the reviewers who provided the listed reports. This is part of the Nature Communications initiative to facilitate training in peer review and to provide appropriate recognition for Early Career Researchers who co-review manuscripts.

(Remarks on code availability)

Open Access This Peer Review File is licensed under a Creative Commons Attribution 4.0 International License, which permits use, sharing, adaptation, distribution and reproduction in any medium or format, as long as you give appropriate credit to the original author(s) and the source, provide a link to the Creative Commons license, and indicate if changes were made.
In cases where reviewers are anonymous, credit should be given to 'Anonymous Referee' and the source.
The images or other third party material in this Peer Review File are included in the article’s Creative Commons license, unless indicated otherwise in a credit line to the material. If material is not included in the article’s Creative Commons license and your intended use is not permitted by statutory regulation or exceeds the permitted use, you will need to obtain permission directly from the copyright holder.
To view a copy of this license, visit https://creativecommons.org/licenses/by/4.0/

REVIEWER COMMENTS & RESPONSES

Reviewer #1 (Remarks to the Author):

This manuscript by Dr. Raj and colleagues is a complex exploration of cellular memory in the context of therapy resistance in cancer cells. The overarching aim is to determine whether resistance to treatments arises from selection and further adaptation to treatment stress, or, in contrast, if resistance is predefined in specific cells within the tumor population. The authors used a single melanoma cell line treated primarily with the BRAFV600E inhibitor vemurafenib as a model system. They provide evidence supporting an adaptive cellular mechanism rather than a pre-existing resistant cell population prior to treatment initiation. Gene expression and ATAC-seq data suggest a primed state of resistance, with minor changes in expression and chromatin accessibility that become more pronounced in resistant cells. Additionally, AP-1 transcription factor binding motifs are enriched in the open chromatin regions in resistant cells. Further experiments suggest that cellular adaptation to the treatment occurs in a dose-dependent manner.

In the second part of the manuscript, the authors investigate whether specific gene expression changes during the adaptation of resistant cells are the result of a predefined gene regulatory program or an acquired adaptation, referred to as "memory," the latter being the proposed mechanism. Finally, they generated cell lines expressing a reporter system with canonical AP-1 binding sites upstream of a minimal promoter that drives expression of either EGFP or mCherry. Using this system, they propose that "the epigenetic memory formed by the addition of trametinib is encoded in cis."

I found this manuscript challenging to read and follow. The text is concise, but the language is occasionally too grandiose, and some conclusions seem overstated. Figure legends are also too brief, often missing important details. While certain aspects of this study are convincing (I particularly appreciate the results in Figure 4d and the experimental approach in Figure 2d), the study's main conclusions are based on experiments conducted in a single cell line and with only one type of treatment. Additionally, the authors do not convincingly demonstrate epigenetic memory as claimed; instead, they merely show that AP-1 sites are accessible, which alone is insufficient to assert that epigenetic memory governs resistance.

> We thank the reviewer for their many helpful comments. We appreciated that they liked some aspects of our experimental design. We also understand why they may have found the manuscript itself hard to follow; we agree that our text was dense, and we have attempted to overhaul it.
>
> The author also made a point about our use of a single cell line. We have now added another cell line to our analysis, a bronchioalveolar carcinoma line (H358) treated with sotorasib (KRAS G12C inhibitor). We have demonstrated that the same adaptive behavior with sotorasib, a KRAS G12C-specific inhibitor (low-to-high drug dose adaptation), and the dexamethasone-induced memory occurs in this cell line as well. The results in this cell line, with a completely different drug and mechanism of action, provide powerful proof of the generality of the concepts in our manuscript, and we thank the reviewer for the suggestion.
>
> Regarding AP-1, we have now provided further data showing that inhibiting AP-1 with multiple drugs inhibits memory formation. Specifically, in addition to our previous JNK inhibition experiments, we tested T-5224, a small molecule inhibitor that blocks the DNA binding activity of c-Fos/c-Jun, providing a mechanistically distinct approach to AP-1 inhibition (Supplemental Figure 3G). We found that co-treatment with 1µM T-5224 and low-dose trametinib (1nM) for two weeks, followed by low-dose trametinib alone, did not substantially affect colony survival compared to low-dose treatment alone.

However, when cells receiving the same T-5224 co-treatment were subsequently challenged with high-dose trametinib (5nM), we observed a significant decrease in final colony numbers, mirroring the effects we saw with the JNK inhibitors. Notably, T-5224 at this dose and duration did not affect the survival of cells exposed directly to high-dose trametinib, indicating that the inhibitor specifically disrupts the adaptive memory process rather than simply increasing drug sensitivity. **Thus, the adaptive effects of drug resistance appear to be specifically dependent on AP-1 activity.**

We have also added further experiments showing that activation of AP-1 has the expected effects. In addition to our previous results, which showed that AP-1 mediated memory of expression upon treatment with trametinib, we found that brief activation of AP-1 with PMA resulted in enhanced retention of our reporter signal, even in the absence of the drug (Supp. Fig. 7D). This result with an AP-1 reporter shows that activation of AP-1 will specifically cause epigenetic memory formation. Together, these results using multiple pharmacological approaches—JNK inhibition, direct AP-1 DNA-binding inhibition, and AP-1 activation—provide strong further evidence for a functional role of AP-1 in both the formation and maintenance of epigenetic memory underlying therapeutic resistance. We thank the reviewer for noting this issue, and we believe our new results greatly strengthen our conclusions.

Honestly, I struggled to fully understand Figure 3, despite reviewing it multiple times over different days. The text does not aid comprehension, and some gene expression differences, such as those of LTBP1 and TGM2, are unclear (e.g., I don't observe their upregulation upon trametinib treatment). Moreover, there is notable variability in FKBP5, LTBP1, and TGM2 between replicates. Along these lines, some conclusions are based on changes in the expression of a single gene (FKBP5) in one cell line and under one treatment condition.

Overall, I find this study to be too preliminary, with conclusions that are largely overstated.

We appreciate the reviewer's many concerns here. We agree that the results of Figure 3 are conceptually dense, and so we reworked the text to make our points clearer. Regarding the other specific points:

Expression differences of LTBP1 and TGM2: We apologize for the confusion our figure caused the reviewer. We point the reviewer to Fig. 3B, Supp. Fig. 5B, D, & E, in which one can see increased expression of these two genes relative to baseline upon treatment with dexamethasone, and their expression returns to baseline 14 days after dexamethasone is removed. Additionally, we would like to highlight that for LTBP1 and TGM2, there is some increase in expression in response to trametinib treatment; however, this increase is relatively small compared to the level of expression of these genes when the gene expression changes from dexamethasone exposure is committed to memory (Fig. 3B, Supp. Fig. 5B, D, & E).

Variability between replicates: The reviewer is right that we observe a lot of variability between replicates. We will say that the trends in each replicate are more replicable, so we could have shown fold changes to make the results more "clean"; however, in the interest of transparency, we elected to show the more "raw" data to ensure that the reader can see the full variability. We believe that the variability is largely due to the often-observed day-to-day variability in cell culture and cell passage number. We have now mentioned that more explicitly in the text, along with some other potential causes.

Single cell line and treatment: As mentioned above, we have now expanded our results to a completely different cancer cell type with a completely different treatment condition (H358 bronchioalveolar carcinoma treated with sotorasib) in Supplemental Figure 4. These results establish the generality of our results, and we thank the reviewer for making this point.

Preliminary/overstated results: We hope that our revised manuscript strengthens our case in the eyes of the reviewer. As mentioned, we have added another cell line from a completely different cancer type. Furthermore, we have added considerable additional mechanistic work on the epigenetic mechanisms of memory formation, in which we outline a key role for p300 and CBP in the maintenance of the epigenetic memory:

> We hypothesized that the encoding of memory in cis could be either established or maintained (or both) through the acetylation of histones by the acetyltransferase (HAT) paralogues p300 and CREB-binding protein (CBP). These enzymes are homologous and function as key transcriptional co-activators that acetylate histone 3 lysine 27 (H3K27ac), a well-established mark of active chromatin at regulatory elements such as enhancers and promoters[39,40]. This acetyltransferase activity is thought to activate gene transcription by creating a more accessible chromatin environment conducive to the binding of transcriptional machinery. The memory formation mechanism we have observed, however, requires that the molecular factor be able to transmit information across cell divisions, maintaining the epigenetic state of the gene as the DNA is replicated. A common mechanism for such information transmission is the bromodomain present in many chromatin-modifying enzymes, such as the Polycomb repressive complex (PRC), although these have been almost exclusively demonstrated for repressive chromatin marks[8,41]. However, recent structural studies using cryo-electron microscopy have revealed that CBP/p300 can act via a "read-write" mechanism[42–44], where the bromodomain reads existing acetylation patterns on histones and the HAT domain writes new acetylation marks, suggesting a mechanism for propagating activating epigenetic memory.
>
> To test our hypothesis, we examined the effects of two distinct inhibitors targeting different functional domains of the CBP/p300 complex both during and after the memory encoding process. We used A-485, a potent, selective, and drug-like catalytic inhibitor that reversibly competes with acetyl-CoA for binding to the HAT domain's catalytic center, thereby directly blocking the histone acetyltransferase activity of CBP/p300 and preventing acetylation of histones and other proteins[43]. To test whether CBP/p300 mediated the epigenetic maintenance of expression, we also used the compound SGC-CBP30, which selectively inhibits the bromodomain of CBP/p300 and prevents the reading of acetylated lysine residues[44,45].
>
> We found that inhibition of both the HAT activity (by A-485) or the bromodomain activity (by SGC-CBP30) of CBP/p300 inhibited the ability of resistant colonies to maintain and amplify the activity of the AP-1 reporter genes. We measured expression of the reporter genes after cells were subjected to trametinib for two weeks, followed by exposure to either A-485 or SGC-CBP30 for an additional two weeks (Fig. 5B-C). In both cases, reporter gene expression went down dramatically, showing that both the activity of the CBP/p300 enzyme and also its bromodomain specifically were required for this form of epigenetic memory. We also subjected the above cells to an additional one week of DMSO treatment to see whether reporter activity would return to the initial levels in the resistant colony. We found that the activity did not recover, suggesting that the memory had been lost (Fig. 5B-C). This result also excludes the possibility of a "*trans*" component to the memory (i.e., transcription factor milieu in the cell), because if the

memory were stored in *trans*, then the memory would recover upon washing out the CBP/p300 inhibitor.

We also tested the effects of concurrent inhibition of CBP/p300 with the MEK inhibition at the start of treatment (Fig. 5D). We found that the inhibition of the HAT activity of the enzyme via A-485 prevented cells from developing into resistant colonies at all. The surviving cells had no discernible fluorescence pattern, and the growth pattern of these cells resembled that of the singletons consistently observed in Goyal et al. 2023. However, concurrent inhibition of the bromodomain activity of CBP/p300 and MEK inhibition via SGC-CBP30 did allow for the formation of resistant colonies, but the AP-1 reporter genes did not increase their activity (Fig. 5D). These results suggest that *de novo* CBP/p300 activity is critical for the formation of resistant colonies, while the bromodomain's putative memory-transfer activity is required for the subsequent maintenance of expression.

Regarding overstatements, we have now made our language more circumspect throughout the revised manuscript. We thank the reviewer for raising these issues, and addressing them has made our paper considerably stronger.

The above results show that cells change their phenotype during exposure to the therapy. A change in cellular state could, in principle, arise either by the execution of a predefined regulatory program, like a predetermined cascade of gene expression changes, or by the encoding of cellular memories specific to the state of the cell at the moment of trametinib exposure. In the latter scenario, the cell could change in ways that were not predetermined by its own in-built regulatory program, thus "learning" how to adapt to therapy. One prediction of the learning hypothesis is that the cell would be able to encode into memory the expression of any gene that was active at the time of adding trametinib, regardless of whether it was part of any putative resistance program or not. Thus, we reasoned that we could distinguish between these two possibilities by forcing a gene to express right at the time of adding trametinib. If the cell executed a prescribed resistance program, then the expression of this gene would return to baseline, whereas if the cell can learn, then the expression would be committed to an epigenetic memory. We thus attempted to plant the memory of expression of a "passenger" gene into cells by expressing the passenger gene transiently at the time of trametinib exposure, then looking for whether that expression was then committed to memory as the cell became fully resistant.

We needed a gene that we could transiently induce to act as the passenger. As an inducer, we used dexamethasone, a steroid that induces expression of a large set of genes through the activation and nuclear translocation of the glucocorticoid receptor[35], and one that is not generally considered to be a major factor in therapy resistance in melanoma (Supp. Fig. 5A). First, we tested whether treating cells with dexamethasone induced genes in a transient manner, meaning that removing the dexamethasone signal would result in a reversion of target gene expression back to baseline levels. Based on bulk RNA sequencing data (Supp. Fig. 5B), we found a set of genes that were induced by dexamethasone, then reverted to baseline when dexamethasone was removed. Of these, we selected three genes (*FKBP5*, *TGM2*, and *LTBP1*) for further quantitative analysis by single-molecule RNA FISH, which enables the counting of transcripts in single cells.

We measured the induction of expression upon the application of dexamethasone and subsequent reversion after dexamethasone removal. We found that each gene was induced upon 3 days of dexamethasone application, returning to baseline after 14 days of growth post-removal. After the reversion time, the cells were quite crowded. To show that the crowding itself was not responsible for the reduced expression after the removal of dexamethasone, we induced cells after crowding to show they were still responsive to dexamethasone (Supp. Fig. 5C). We also split the cells to lower densities after removal and found that expression was still at baseline levels (Supp. Fig. 5D).

Per our hypothesis, if one of these genes were expressed at the point in time that trametinib was applied, its expression would be committed to memory and thus retained, even in the absence of the originating dexamethasone signal. We thus applied trametinib after three days of dexamethasone treatment, at which point we also removed dexamethasone. We measured expression in the resultant resistant colonies after 14 days. For *FKBP5*, we found that the expression levels were as high, if not higher, than with dexamethasone alone after 3 days, even though dexamethasone was removed 14 days earlier. Importantly, the addition of trametinib alone did not induce *FKBP5*, showing that the expression of this gene was not inherently associated with therapy resistance; i.e., not a part of any pre-defined resistance “program”(Fig. 3B). The persistent increased levels of *FKBP5* expression validate our hypothesis that the expression of this unrelated gene has been committed to memory upon trametinib treatment.

For *TGM2* and *LTBP1*, we found a different pattern of expression, albeit one that also indicated memory formation. For these genes, adding trametinib alone did induce expression in the resistant colonies (Fig. 3B). However, treatment with dexamethasone for 3 days beforehand led to stark 4- and 5-fold increases in expression of *TGM2* and *LTBP1*, respectively. This induction level was well beyond that of either trametinib or dexamethasone alone. Thus, for these two genes, the induction of expression was still “memorized” in that without trametinib, the expression level would have returned to baseline, and the expression level was far beyond what trametinib would have induced alone. For all three genes, the expression level at the time of trametinib exposure was committed to memory and affected its future expression, the hallmark of memory and learning. (We noted significant variability between independent biological replicates, likely due to biological variability inherent to cells grown in culture in slightly different conditions. For *TGM2* and *LTBP1*, we found that longer durations of induction resulted in more consistent induction and showed the same memory effect (Supp. Fig. 5D).)

One possible alternative was that once cells become resistant, their induction and reversion timescales upon additional and removal of dexamethasone were different from those of the therapy-naive cells. To test this possibility, we took a population of resistant cells that had never been treated with dexamethasone before and induced them with dexamethasone for 3 days, then cultured them for 7 more days without dexamethasone (Supp. Fig. 5F). We found that the expression of *FKBP5*, *TGM2*, and *LTBP1* all increased upon dexamethasone induction and reverted to baseline levels of expression after dexamethasone was removed. These results show that the mechanism by which these genes are induced is not intrinsically altered by therapy resistance, nor is the sustained expression of these genes merely a consequence of the difference in the regulatory state of resistant cells. Rather, these results show there is a window of opportunity for memory formation during the early period of the addition of trametinib, consistent with our other results.

Reviewer #2 (Remarks to the Author):

The manuscript presents an elegant and thoughtfully designed study on cellular adaptation, a novel and intriguing perspective in the field of cancer therapy resistance. This work contributes significantly to the shifting away from the traditional binary model of resistance, often attributed to selection of pre-existing or acquired resistant cells. The authors elegantly demonstrate the adaptive process where cells respond dynamically to therapy by incorporating previous experiences (drug exposure) into epigenetic memories.

The use of dual-color reporter experiments is particularly creative and compelling, reinforcing the concept of adaptive cellular behavior in a nuanced manner. The finding that the transcription factor AP-1 plays a central role in regulating these adaptive behaviors and establishing a "memory" under therapeutic stress adds an intriguing layer of mechanistic understanding, and aligns well with previous work in cancer and other fields.

Technically, the manuscript is robust, and the experiments are logically arranged, well executed, and thoughtful in design. This paper holds significance for the field and is worthy of publication. Below are specific issues that should be addressed in terms of contextualization and clarity.

> We thank the reviewer for their kind a positive assessment of our work, and also for their careful reading of our manuscript. We believe that by addressing the comments, the manuscript has improved dramatically.

Regarding potential mechanisms for chromatin modifications: a recent study has shown at single-cell resolution and lineage barcoding that cells initially surviving therapy ('persisters') are primed with H3K4me3 and H3K27me3, thus enhancing the potential for resistance (Marsolier et al. Nat Genetics, 2022: PMID: PMC7612638). This could be mentioned in the discussion as possible cis- epigenetic signals. Also, our own study, (França et al., Nature, 2024: PMID: 38987605) used a dose escalation approach, as employed here, and showed that previous exposure to lower doses enabled the emergence of resistant populations to higher doses in an ovarian cancer model — a phenotypic observation that parallels this work. This could be acknowledged and discussed in support of the authors' findings. In addition, transcriptional and chromatin changes that do not resemble initial states ('persister' cells) with increased AP-1 accessibility were associated with higher resistance in this work, suggesting recall of an epigenetic memory after drug holiday and re-exposure. In the present study, the authors have uniquely demonstrated some of these processes with greater resolution and detail using single-clone tracking and cellular barcoding, with very interesting results. Addressing these recent findings would provide support and contextualization.

> We thank the reviewer for pointing out these important studies, especially their own work, França et al., Nature, 2024, which we agree is highly relevant. We have now updated our text to incorporate these references and discussion points.

>> Introduction
>> …
>> Here, we demonstrate in a model of therapy resistance that cells display various characteristics of cellular memory formation and learning. They show an adaptive phenotypic behavior, exhibiting the ability to resist higher doses of therapy than pure selection would predict, as has been recently shown also by França et al.[27]. They also show the ability to encode the memory of the transcriptional state of the cell at the time of therapy application, maintaining and even

enhancing the expression of otherwise transiently expressed genes (both induced and stochastically expressed). We demonstrate that the transcription factor AP-1 is an important mediator of this memory effect. Further, we show that AP-1 reporter genes encode memories in *cis*, meaning that the memory is encoded based on the instantaneous activity of the gene and not the DNA sequence alone. Together, our results establish the formation and maintenance of cellular memories as an important aspect of gene regulation in therapy resistance and suggest molecular mechanisms that may be responsible for cellular memory and learning in other biological contexts as well.

Discussion

…

One consequence of stress-induced memories is the potential for the accidental formation of deleterious memories. A cell, upon encountering stress, may form a memory of transiently expressing a gene whose expression is in some way incompatible with or otherwise harmful to the cell. The accumulation of deleterious memories may lead cells to function less well over time, constituting a mechanism for the generation of epigenetic damage during aging. Removing such memories from cells could potentially restore their prior functionality. In the context of therapy resistance in cancer, removing such memories may cause resistant cells to revert to a cellular state that is now vulnerable to either the original therapeutic agent or some new set of agents.

Our results on dose escalation demonstrate that cells can actively adapt to treatment, mirroring the work of França et al.[27], which also found that adaptation to low doses of therapy can lead to resistance to higher doses. Together, these results suggest that adaptation may be a common mechanism of therapy resistance, with important consequences for treatment regimens.

We have here drawn a distinction between programmed and learned responses to stimuli (*trans* vs. *cis* epigenetics, respectively). In the most general terms, the distinction is artificial in the sense that even responses based on learning can be thought of as the execution of a program. We think a more philosophically coherent description is to consider the mapping between the inputs to and outputs from the cell. Program execution corresponds to what could be considered an input funnel, in which many different inputs lead to the same output (say, a developmental outcome as a result of a regulatory cascade). Learning is more akin to a one-to-one mapping, in which each different input leads to a different output. Such output contingency is a hallmark of learning and is a practically useful distinction.

In Figure 1, further clarification is needed regarding the transcriptomic data. Specifically, while the authors cite their 2021 paper, additional details on how the transcriptomic and chromatin profiles of the ‘non-primed’, ‘primed’ and ‘resistant’ cells were defined a priori. A better clarification on how these populations were defined is required.

We agree completely and apologize for the lack of clear definitions. We have now added these definitions to our introduction for clarity.

Introduction

…

Here, we develop the concept of cellular learning in the context of therapy resistance in cancer cells. Typically, therapy resistance is thought to be driven by genetic differences, which would suggest a change to the hard-coded regulatory framework of the cell. Work from our lab and others, however, has shown that therapy resistance can arise from rare “primed” cells with non-genetic differences[16–26], with primed cells being defined as cells that can survive therapy and non-primed cells being the ones that die. The existence of non-genetic differences suggests different possibilities for the acquisition of therapy resistance: cells primed for resistance could simply be selected by the therapy (as per natural selection of existing variation), or cells primed for resistance could change in some adaptive fashion, ultimately arriving at a fully therapy-resistant fate that is different from the initial primed state (fully resistant meaning that they remain resistant even after prolonged drug holiday[16]). Our prior results suggest the latter scenario, with both molecular and phenotypic differences between primed and fully resistant cells. Although this possibility is consistent with other recent results[27], a definitive demonstration of cellular adaptation has proven challenging.

…

Results

To probe the potential for cellular learning, we used a cellular model for non-genetic resistance to cancer therapy. Specifically, we used a melanoma cell line WM989[28] in which the vast majority of cells die upon MAPK pathway inhibition (using either the $BRAF^{V600E}$ inhibitors vemurafenib/dabrafenib or the MEK inhibitor trametinib). Despite having clonally bottlenecked the cell line, however, a small fraction (around 1 in 2000) of the cells (which we dub “primed” for resistance) survive the therapy treatment and resume proliferation after a short period of quiescence, ultimately forming a therapy-resistant colony in cell culture. (Note that these colonies are generally considered “fully resistant” in that they retain their resistance even after an extensive drug holiday[16].) We have assembled multiple lines of evidence that there is no genetic basis for these rare cells becoming therapy-resistant, including Luria-Delbrück fluctuation analysis[16] and DNA sequencing (both targeted panel[16] and whole genome sequencing[20] of multiple therapy-naive and therapy-resistant clones). Instead, we have demonstrated that the rare-cell expression of certain sets of genes is strongly associated with cells being primed for therapy resistance[16,18]. The therapy induces a strong selection for survival or death based on the state of the cell (primed or not primed, respectively) before the therapy is initiated, but the primed cells could follow different trajectories as therapy treatment continues. One possibility is that the primed cell is selected by the therapy and expands but does not change; i.e., the primed cell is the same as the resistant cell. Such a scenario is typically the case in instances of the selection of genetic mutants. However, another possibility is that primed cells undergo a transformation during their time in therapy akin to cellular adaptation, such that the initially primed cell changes to become the fully therapy-resistant cell over time (Fig. 1A).

…

‘Full resistance’ could be better defined in this context. Do the authors consider ‘resistant’ as cells that are able to survive drug exposure, to proliferate under treatment, or through a quantitative measure such as the half-maximal inhibitory drug concentration (IC50)? A more explicit and, if possible, quantitative definition of resistance in this model would provide additional clarity.

The reviewer has made an excellent point about “full” resistance. We define “full resistance” as the ability to remain resistant even after the drug exposure is removed (drug “holiday”) and then reapplied later. We have now updated the text accordingly.

To probe the potential for cellular learning, we used a cellular model for non-genetic resistance to cancer therapy. Specifically, we used a melanoma cell line WM989[28] in which the vast majority of cells die upon MAPK pathway inhibition (using either the $BRAF^{V600E}$ inhibitors vemurafenib/dabrafenib or the MEK inhibitor trametinib). Despite having clonally bottlenecked the cell line, however, a small fraction (around 1 in 2000) of the cells (which we dub "primed" for resistance) survive the therapy treatment and resume proliferation after a short period of quiescence, ultimately forming a therapy-resistant colony in cell culture. (Note that these colonies are generally considered "fully resistant" in that they retain their resistance even after an extensive drug holiday[16].) We have assembled multiple lines of evidence that there is no genetic basis for these rare cells becoming therapy-resistant, including Luria-Delbrück fluctuation analysis[16] and DNA sequencing (both targeted panel[16] and whole genome sequencing[20] of multiple therapy-naive and therapy-resistant clones). Instead, we have demonstrated that the rare-cell expression of certain sets of genes is strongly associated with cells being primed for therapy resistance[16,18]. The therapy induces a strong selection for survival or death based on the state of the cell (primed or not primed, respectively) before the therapy is initiated, but the primed cells could follow different trajectories as therapy treatment continues. One possibility is that the primed cell is selected by the therapy and expands but does not change; i.e., the primed cell is the same as the resistant cell. Such a scenario is typically the case in instances of the selection of genetic mutants. However, another possibility is that primed cells undergo a transformation during their time in therapy akin to cellular adaptation, such that the initially primed cell changes to become the fully therapy-resistant cell over time (Fig. 1A).

A number of lines of evidence suggest the latter scenario—that resistant cells transform during therapy treatment. In our previous work, at the phenomenological level, we have shown that primed cells are only transiently primed[16,17,29] while fully resistant cells remain resistant for months in continuous culture[16,20]. This demonstrated that cells change their phenotype between being primed and becoming fully resistant. To see how such changes manifested at the molecular level, we reanalyzed the transcriptomes of non-primed, primed, and fully resistant cells by retrospective identification of primed cells[18] (Fig. 1B-D). As in Shaffer et al. (2017)[16,18] and Emert et al. (2021)[16,18], we found that while primed cells certainly showed important differences in their transcriptome profile from non-primed cells, fully resistant cells differed far more dramatically. The expression of key priming marker genes (e.g. *AXL, ITGA3, SERPINE1*) was all considerably higher in resistant cells as compared to primed cells. These results further suggested that primed cells undergo an adaptive process as they become fully therapy-resistant.

To find the regulatory factors that may be involved in this adaptive process, we measured changes in chromatin accessibility as cells became fully therapy-resistant using ATAC-seq[30,31]. We found that there were 9197 peaks that had higher accessibility in primed cells compared to non-primed cells, compared to 15,627 peaks with increased accessibility in resistant cells (defined as cells surviving after 4 weeks of treatment) compared to non-primed cells. For 1 week post-treatment, there were 4722 peaks compared to non-primed cells, and 1901 of those peaks were shared with primed cells[16]. We thus conclude that while there were a few peaks that gradually increased their accessibility, many were only open in the primed state. A reanalysis of the motifs in these loci showed enrichment for binding sites for a number of transcription regulators, notably the general transcriptional regulator AP-1 (Fig. 1E). AP-1, which is generally composed of a heterodimer of FOS and JUN subunits, is often identified in ATAC-seq data and disregarded due to its ubiquity. However, our hypothesis is that when a cell transitions from primed to fully resistant, the changes in gene expression are not exclusively predetermined by

hard-coded regulatory programming. We reasoned that the very ubiquity of AP-1 would allow it to potentially reprogram cells in a flexible manner, depending on the current state of the cell, rather than any pre-existing regulatory program.

In several figures showing ATAC-seq profiles (ex: Fig. 1e) the colorbar is not labeled.

We thank the reviewer for pointing out these issues; they are now fixed.

Reviewer #3 (Remarks to the Author):

The paper by Li, et al continues the Raj group's in-depth investigation of the cellular resistance and memory effect in models of cancer. Here the focus is on buildup of cellular memory and the involvement of AP-1 in mediating it. The authors' model suggests that following stress (in this paper it is drug exposure), gene expression that may have been linked with exposure, but also transient or incidental, can become fixated through a mechanism that is dependent on AP-1 activity. Such expression can be stably propagated in affected clones. It may can lead to adaptation, for example in scenarios of gradual dose increase. And it is shown to be facilitated in-cis, according to experiments separating clonal memory effects in two independent loci.
This is a really interesting, original and important work. The experimental design, data collected and analysis are all of top quality. I strongly support its timely publication.

We thank the reviewer for their kind and positive assessment. We are also grateful for their many useful suggestions and believe that by addressing them, our manuscript has been greatly improved.

I'm listing here some points for potential consideration by the authors. These can be used to improve presentation/clarity and add some potential insights, but I do not believe the paper should be expanded significantly or that new data should be added. The questions addressed here are going to be followed up by the authors and by others, and the present study represent an important advance. I provide some suggestions for adding evidence on the mechanisms for in-cis memory using more ATAC analysis, but in my opinion, if the authors decide they wish to keep the arguments here at a higher level, then this can be accepted.
Points for consideration:

We really appreciate the reviewer's comments about scope; we also feel that the scope creep of peer review has had a very negative effect on the efficiency of the scientific enterprise. We have addressed the ATAC-seq points below.

1) Primed, resistant and adapted cells. The paper is based on a rather sophisticated, and potentially difficult to understand distinction cell priming and adaptation. These are complementary ideas which may be perceived as contradicting (e.g. one student asked: if only primed cells can adapt, is this really adaptation..?). The authors schematics in Figure 1 is perhaps not highlighting the most important aspects of this distinction, as it seems that the "adaptation" phase is just a direct consequence of the original response. It should be better clarified – a) that the altered state can persist after removing the stimulation. b) that it is possible that not all "primed" cells can adapt. If the author can further come up with a more uniform schematics for the experiments in Figure 2-3, it would be very helpful.

The reviewer has made an excellent point about our definitions, and we agree that our initial presentation did not precisely and intuitively define these concepts. This point was also brought up by reviewer 2. We have now expanded our discussion of these definitions in the introduction, and have briefly re-explained the concepts in the main text as well. We have also updated the schematics to be more consistent and hopefully more understandable.

Introduction

…

Here, we develop the concept of cellular learning in the context of therapy resistance in cancer cells. Typically, therapy resistance is thought to be driven by genetic differences, which would suggest a change to the hard-coded regulatory framework of the cell. Work from our lab and others, however, has shown that therapy resistance can arise from rare "primed" cells with non-genetic differences[16–26], with primed cells being defined as cells that can survive therapy and non-primed cells being the ones that die. The existence of non-genetic differences suggests different possibilities for the acquisition of therapy resistance: cells primed for resistance could simply be selected by the therapy (as per natural selection of existing variation), or cells primed for resistance could change in some adaptive fashion, ultimately arriving at a fully therapy-resistant fate that is different from the initial primed state (fully resistant meaning that they remain resistant even after prolonged drug holiday[16]). Our prior results suggest the latter scenario, with both molecular and phenotypic differences between primed and fully resistant cells. Although this possibility is consistent with other recent results[27], a definitive demonstration of cellular adaptation has proven challenging.

…

Results

To probe the potential for cellular learning, we used a cellular model for non-genetic resistance to cancer therapy. Specifically, we used a melanoma cell line WM989[28] in which the vast majority of cells die upon MAPK pathway inhibition (using either the $BRAF^{V600E}$ inhibitors vemurafenib/dabrafenib or the MEK inhibitor trametinib). Despite having clonally bottlenecked the cell line, however, a small fraction (around 1 in 2000) of the cells (which we dub "primed" for resistance) survive the therapy treatment and resume proliferation after a short period of quiescence, ultimately forming a therapy-resistant colony in cell culture. (Note that these colonies are generally considered "fully resistant" in that they retain their resistance even after an extensive drug holiday[16].) We have assembled multiple lines of evidence that there is no genetic basis for these rare cells becoming therapy-resistant, including Luria-Delbrück fluctuation analysis[16] and DNA sequencing (both targeted panel[16] and whole genome sequencing[20] of multiple therapy-naive and therapy-resistant clones). Instead, we have demonstrated that the rare-cell expression of certain sets of genes is strongly associated with cells being primed for therapy resistance[16,18]. The therapy induces a strong selection for survival or death based on the state of the cell (primed or not primed, respectively) before the therapy is initiated, but the primed cells could follow different trajectories as therapy treatment continues. One possibility is that the primed cell is selected by the therapy and expands but does not change; i.e., the primed cell is the same as the resistant cell. Such a scenario is typically the case in instances of the selection of genetic mutants. However, another possibility is that primed cells undergo a transformation during their time in therapy akin to cellular adaptation, such that the initially primed cell changes to become the fully therapy-resistant cell over time (Fig. 1A).

…

2) Describing complex experiments. I believe more subheadings/sections in the results, breaking the experimental work into smaller chunks with clear conclusions for each step, can be very helpful. The current

experimental narrative is beautiful, but require quite some effort for following the complex logic in relatively long series of arguments – it is unclear if typical readers will naturally go through this.
I did like the usage of additional text to explain supplementary figures (beyond legends). I hope this can be accommodated by the journal guidelines – it is the right thing to do!

> The reviewer has made a great suggestion. We have adopted it and have tried to break up the narrative with subheadings that make it easier to follow the admittedly complex logic.

3) In cis-memory and ATAC. ATAC analysis can probably provide significantly more insight. ChromVAR motif plots in Figure 1 and Figure S4 only show broad statistical behaviors that can be linked with (families of) TFs. Identifying accessibility peaks and clustering these to identify the main modes in the data can provide a much more direct read out. I realize this may be a domain the authors wish to follow up thoroughly later, which I can understand and support, but showing the ATAC data without even minimal in-cis analysis seems at odds with the very systematic approach to experiments that is represented in all other parts of this study. Possible points for consideration (3.1 seems like a must, the others optional at this stage):

> We agree with the reviewer completely and have performed the insightful analyses they have suggested, with details given below.

3.1 Quantitative vs. hierarchical epigenetic programming – are there specific clusters of loci (not motifs!) that are becoming accessible only following adaptation, and clusters that become accessible already in the primed state, such that the average generate the gradual motif footprints shown now? Or is it gradual at the level of individual loci?

> We have performed a peak-centric analysis on the ATAC dataset in Figure 1. We classified the conditions as EGFR negative (not primed), EGFR positive no drug (primed), EGFR positive 1 week of drug (adapted), and EGFR positive 4 weeks of drug (resistant). We then compared the peaks that have increased accessibility compared to not primed and checked which ones were consistent across conditions and which were unique to particular ones (Supp. Fig. 1A,B). We found that 5.5% of the peaks were already more accessible in the primed condition and stayed open all the way through resistance, while many other peaks were unique to particular conditions. Interestingly, there was a higher degree of overlap between primed and resistant (16.3%) as opposed to primed and adapted (3.1%) or resistant and adapted (4.1%).

3.2 TSS, proximal, distal behaviors – would adaptation occur mostly at TSSs (probably not)? If it is also detected away from TSSs, are there specific chromosomal domains showing adaptation? For loci with potential AP-1 in-cis specificity, would the chromosomal context be distributed differently?

> We have added chromosomal context analysis around peaks from the ATAC dataset to Figure 3 (Supp. Fig. 6E). We classify each dex-responsive peak (defined as being uniquely accessible in the 3 day Dex condition compared to 14 days of tram) as either Learned or not Learned, depending on whether or not they stay high in the dex → tram condition and with or without AP1 motifs. We then generate random subsets of the dex-specific peaks corresponding to the sample size of the aforementioned peak sets. For each of these, we run annotation analysis of all of these, and broadly we find no differences across these peak sets (Supp. Fig. 6E).

3.3 linkage to gene expression and feedback – How many of the adapted hotspots can be reasonably linked with a transcriptionally regulated TSS? (e.g. are within the same TAD or within e.g. 100kb)? Can the authors identify a group of ATAC peaks that are particularly isolated from any discernible transcriptional regulation?

This will be really important and interesting in the context of feedback memory vs. in-cis transmission – concepts that are discussed a bit in the paper, but without much evidence.

> We took each peak from the analysis described in 3.2 and quantified the distance to the nearest TSS. We found that ~75% of all of the peaks for each were found within 100kb of TSS. Interestingly, we found that 17% of the Learned with AP1 peaks were within 10kb of the TSS, while 20% of Learned without AP1 peaks and Not Learned with AP1 peaks were in the same range. 26% of the not learned with AP1 peaks were within 10kb of the TSS (Figure 3E,F).

3.4 Activated and repressive chromatin. One should identify TSSs or regulatory elements that are losing accessibility during adaptation/selection. If there are very few, it should be quantified. If not, this should be further characterized and discussed. Points for consideration: linkage with transcription, gradual decay, and the transmission/propagation of these changes in resistant clone (is repression only transient, and cannot be memorized? Is it linked with AP-1? An indirect consequence of Ap-1 mediated activation? If the argument is that there are _no_ accessibility hotspots that are being decreasing in intensity following adaptation, it will be quite surprising and need some better statistical quantification.

> Similar to the analysis performed in 3.1, we took the primed, adapted, and resistant peak sets and found ones that lose accessibility compared to not primed cells. We found that of these peaks, 21.6% were already lost in primed and remained inaccessible throughout resistance. Again, we found many peaks that were unique to each of the conditions, with 29.6% of these lost peaks unique to the resistant cells (Supp. Fig. 1A,C).

Minor atac points:
- The color scale of chromvar should be labled.
- Enrichment of CTCF motifs is hard to explain
- IT will be helpful to keep the rows (motifs) in the analysis using the same order across Fig 1 and S4 panels – for comparison.

> We thank the reviewer for pointing out these issues; appropriate labels have been added to the chromvar scale bar. For Fig. 1 and S4 panels. For the point about the row ordering, while we appreciate the suggestion, these come from different experiments, so we felt it most appropriate to unbiasedly rank them by top variable motifs.
>
> We thank the reviewer for this important observation on CTCF enrichment. We believe that the enrichment of CTCF motifs in trametinib-resistant cells may reflect chromatin reorganization during the development of resistance, and possibly that CTCF binding at these sites would help establish and maintain the newly opened chromatin architecture, creating stable regulatory domains that support the resistant state. The depletion of CTCF motifs with JNK inhibition matches this model. The intermediate CTCF enrichment with dexamethasone followed by trametinib treatment may reflect a complex interplay between glucocorticoid signaling and chromatin architecture; our data are too limited to make any conclusions, but we agree that it is an interesting finding.

4) proliferation and memory decay. It would be important to understand better memory decay and the directions to stress as analyzed here. If clones do maintain an adapted state for a month as briefly mentioned here, is it suggested “switching back” to the ground state is sufficiently rare to be ignored? What is the degree of stochasticity within the adapted clonal state, and can the memory be eliminated by another stress? These

are points for discussion, mostly important in order to understand whether homeostasis or directional adaptation is the main mode to be expected within a tumor cell population,

Thank you for this fantastic suggestion. It is something we have wondered about for quite some time. To address the question of how long the memory seems to persist, we performed another burn-in experiment with the dexamethasone to trametinib condition. Instead of a14 day duration, we waited for 28 and 35 days before performing FISH for key dexamethasone-responsive genes (FKBP5 and TGM2). At these timepoints, the cells that experienced dexamethasone prior to trametinib still had high numbers of transcripts for the dexamethasone-responsive genes (Supp. Fig. 5G), showing that the memory was retained for a long time period.

Reviewer #4 (Remarks to the Author):

I co-reviewed this manuscript with one of the reviewers who provided the listed reports. This is part of the Nature Communications initiative to facilitate training in peer review and to provide appropriate recognition for Early Career Researchers who co-review manuscripts.
